# Headbobber: A Combined Morphogenetic and Cochleosaccular Mouse Model to Study 10qter Deletions in Human Deafness

**DOI:** 10.1371/journal.pone.0056274

**Published:** 2013-02-14

**Authors:** Annalisa Buniello, Rachel E. Hardisty-Hughes, Johanna C. Pass, Eva Bober, Richard J. Smith, Karen P. Steel

**Affiliations:** 1 Wellcome Trust Sanger Institute, Hinxton, Cambridgeshire, United Kingdom; 2 Wolfson Centre for Age-Related Diseases, King's College London, London, United Kingdom; 3 MRC Institute of Hearing Research, Nottingham, United Kingdom; 4 Max-Planck-Institute for Heart and Lung Research, Bad Nauheim, Germany; Indiana University School of Medicine, United States of America

## Abstract

The recessive mouse mutant headbobber (*hb*) displays the characteristic behavioural traits associated with vestibular defects including headbobbing, circling and deafness. This mutation was caused by the insertion of a transgene into distal chromosome 7 affecting expression of native genes. We show that the inner ear of *hb/hb* mutants lacks semicircular canals and cristae, and the saccule and utricle are fused together in a single utriculosaccular sac. Moreover, we detect severe abnormalities of the cochlear sensory hair cells, the stria vascularis looks severely disorganised, Reissner's membrane is collapsed and no endocochlear potential is detected. Myo7a and Kcnj10 expression analysis show a lack of the melanocyte-like intermediate cells in *hb/hb* stria vascularis, which can explain the absence of endocochlear potential. We use Trp2 as a marker of melanoblasts migrating from the neural crest at E12.5 and show that they do not interdigitate into the developing strial epithelium, associated with abnormal persistence of the basal lamina in the *hb/hb* cochlea. We perform array CGH, deep sequencing as well as an extensive expression analysis of candidate genes in the headbobber region of *hb/hb* and littermate controls, and conclude that the headbobber phenotype is caused by: 1) effect of a 648 kb deletion on distal Chr7, resulting in the loss of three protein coding genes (*Gpr26*, *Cpmx2* and *Chst15*) with expression in the inner ear but unknown function; and 2) indirect, long range effect of the deletion on the expression of neighboring genes on Chr7, associated with downregulation of *Hmx3, Hmx2* and *Nkx1.2* homeobox transcription factors. Interestingly, deletions of the orthologous region in humans, affecting the same genes, have been reported in nineteen patients with common features including sensorineural hearing loss and vestibular problems. Therefore, we propose that headbobber is a useful model to gain insight into the mechanisms underlying deafness in human 10qter deletion syndrome.

## Introduction

Numerous mouse mutants with hearing defects and vestibular problems are available as models for understanding human deafness, many of them arising from different mutagenesis programs [1], [2]. More than 300 human syndromes with the presence of deafness and/or vestibular malfunction due to abnormal inner ear development have been described to date [3], and still a lot of work needs to be done in order to identify the causative genes for a number of these pathologic conditions.

Here we describe headbobber (*hb*), a recessive mouse mutant created by insertional mutagenesis in a transgenic line carrying a 8 kb plasmid phβAPr-1neo, which includes a portion of the human beta actin promoter [4]. Approximately 2 weeks after birth, the headbobber homozygous mutants start to show abnormal hyperactivity and circling movements which are typical of balance defects [5]. Moreover, they are completely deaf. The main features of the *hb/hb* inner ear phenotype include strial abnormalities with lack of intermediate cells, lack of interdigitations between marginal and basal cells and collapse of Reissner's membrane, which is sufficient to explain the absence of endocochlear potential [6]–[8]. The *hb/hb* vestibular system shows severe morphological defects, with lack of semicircular canals and cristae together with the formation of a characteristic fused utriculo-saccular compartment hosting a fused macula. We have been able to localise the headbobber mutation to an 8 Mb region on the distal portion of mouse chromosome 7 on an intraspecific backcross ([*hb*/*hb* X CBA] F_1_ X *hb*/*hb*). The transgene was mapped to the same region of chromosome 7, 65 cM from the centromere between the markers *D7Mit105* and *D7Mit12*, on a reciprocal backcross ([*hb*/*hb* X CBA] F_1_ X CBA). We have used microarray Comparative Genomic Hybridization [9] and whole genome sequencing to narrow down the headbobber region, and found a 648 kb homozygous deletion in the distal part of mouse chromosome 7F3 500 kb telomeric to the *Hmx3* and *Hmx2* locus [10]. We have previously reported in published abstracts the head bobbing and circling behavior, deafness, failure in semicircular canal formation, mapping of the phenotype and the transgene to chromosome 7 by linkage analysis, and non-complementation with *Hmx3* [11]–[13] and present the full dataset here. While this manuscript was in the final stages of preparation, a paper describing the same mutation and general phenotype in the headbobber mouse was published [4], where the authors utilise molecular, histological, electrophysiological and genomic tools to characterize the headbobber inner ear phenotype, identify the mutation and map the transgene integration site.

In this work, we propose a mechanism to explain both the hearing and vestibular phenotypes shown by the headbobber mutant and link it to the expression pattern in the inner ear of the three deleted genes (*Gpr26*, *Cpxm2* and *Chst15*). Interestingly, we report that different-sized deletions in the homologous region in humans have been reported in fifty-nine patients affected by the so-called 10qter syndrome [14]–[18]. These patients display a wide clinical variability of features which include sensorineural deafness and vestibular defects in nineteen cases [18]. Therefore, we propose that headbobber is a useful model to gain insight into the mechanisms underlying human 10qter deletions and to identify the candidate genes for this disease.

Furthermore, we use the headbobber mouse model to investigate the overlapping role of the homeobox transcription factors *Hmx2*, *Hmx3* and *Nkx1-2* in inner ear development and to gain more insights on their transcriptional regulation [19].

## Materials and Methods

### Ethics statement

All mouse breeding and investigation were carried out with authorization of the UK Home Office project license. All mice were killed by cervical dislocation and decapitation, all surgeries were performed under anesthesia with urethane (2 mg/kg). All efforts were made to minimize suffering.

### Mouse mutants

Headbobber was created by transgenic insertion arising in a transgenic line carrying a 8 kb plasmid phβAPr-1neo, which includes a portion of the human beta actin promoter [4], [20]. Mice carrying the headbobber mutation were originally obtained from Paul Overbeek at the Baylor College of Medicine, Houston, Texas. Details of the genetic background were unknown, but the mutants were maintained within a closed colony and heterozygote or wildtype littermates were used as controls. The *Hmx3^KO^* mice were described in [20], (MGI name: *Hmx3^tm1Ebo^*). All mouse breeding and investigation were carried out with authorization of the UK Home Office.

### Phenotyping and behavioural analysis

A brief 20 kHz soundburst at the intensity of 90–100 dB was generated by a custom-made click box. A flick backwards of the pinna upon hearing the sound was counted as a positive Preyer response. Other behavioral testing (air righting, contact righting, Elevated Platform, Negative Geotaxis and reaching and swimming behavioral tests) were performed as described previously [11].

### Genetic mapping

Intraspecific backcrosses were generated by crossing head bobber mice with the inbred strain CBA/Ca. To map the mutation, the following backcross was used (*hb/hb* X CBA/Ca)F_1_ X *hb/hb*. Genomic DNA was examined using PCR primers flanking microsatellite regions. A subset of 34 of the backcross mice were used for a genome scan (data not shown). Only 11% recombination was found between the headbobber phenotype and *D7Mit9*. The rest of the backcross mice were typed for this marker and 7 other chromosome 7 markers (Figure S1). The headbobber mutation cosegregated with *D7Mit241* and *D7Mit71* and was proximal to *D7Mit12* and distal to *D7Mit103* and *D7Mit105*. To map the transgene, the backcross (*hb/hb* X CBA/Ca)F_1_ X CBA/Ca was used. To determine the presence or absence of the transgene, primers to the neomycin resistance component were used. The mice generated to map the transgene were analysed for the markers *D7Mit105* and *D7Mit12* and typed for the presence or absence of the transgene.

### Genotyping and Neo counting

srPCR was run to confirm the absence/presence of Neo and absence/presence of a region deleted in the *hb* mutant. Primers used were Neo F (CAAGATGGATTGCACGCAGGTTCTC), Neo R (GACGAGATCCTCGCCGTCGGGCATGCGCGCC), hb F (CACAACGTCGATGAGTTATTTAG) hb R (ACTATCACAACCACCAGCAGC).

Neo F and Neo R produced a product approximately 550 bp, hb F and hb R produced an approximately 120 bp product. Presence of *hb* and absence of Neo indicated a wildtype, absence of *hb* and presence of Neo indicated a homozygous mutant, and presence of both indicated a heterozygote.

qPCR was run to calculate number of copies of Neo, and thus the transgene, present in the *hb* mice. This was done using the standard Sanger Institute protocol, details of which can be found at http://www.knockoutmouse.org/kb/entry/91/. Accessed 2012 Aug 2.

### Complementation test

*hb/hb* mice on the original genetic background were crossed with mice heterozygous for the *Hmx3^KO^* [20]. The presence of the knockout allele was typed by Southern blotting using a 1090 bp EcoRI/StuI fragment of the *Hmx3* cDNA. The *hb* allele generated a band of 9070 bp and the knockout allele generated a band of 4911 bp. The offspring (*Hmx3^KO^/hb* N = 12, *+/hb* N = 9) were tested for the Preyer reflex, the swimming test, the reaching response, the air righting response, the contact righting response, the elevated platform test, negative geotaxis and compared with the *Hmx3* knockout mice (*Hmx3^KO^/Hmx3^KO^* N = 7, +/*Hmx3^KO^* N = 5).

### Endocochlear Potential measurements

Endocochlear potential from *hb/hb*, *+/hb*, *Hmx3^KO^/Hmx3^KO^*, +/*Hmx3^KO^*, *Hmx3^KO^;hb* and wildtype mice aged 60 to 82 days were measured as previously described [21]. Sex and coat colour show no relationship to the auditory phenotype.

### Compound Action Potentials measurements

Mice were anesthetized with urethane (2 mg/kg), a tracheal cannula was inserted, and the mouse was placed on a heated blanket. The middle ear was opened leaving ossicles intact and a teflon-coated silver wire was placed on the round window of the cochlea. Stimuli were presented via a closed, calibrated sound system and consisted of tone pips of 15 msec duration, 1 msec rise/fall time. Responses to 200 stimuli were averaged and used to determine the threshold, defined as the lowest intensity to produce a recognizable waveform using either 5 dB or 2 dB intensity steps for each frequency tested [22].

### Paintfilling

Nine littermates at postnatal day 1 (three *+/+* controls, three *+/hb* and three *hb/hb*) were fixed in Bodian's Fixative for 60–120 min at 4°C in a rotator. The half heads were processed and filled as previously described [23]. Ears were viewed with a Leica MZ16 light microscope and images were acquired by the Leica DFC490 camera.

### Scanning Electron Microscopy

A total of 10 mice aged P5 (3 *hb/hb* 4 *+/hb* and 3 *+/+*) were investigated by scanning electron microscopy (SEM) using the OTOTO method, as previously described [24], and viewed with a Hitachi FE S-4800 Scanning Electron Microscope operated at 3–5 kV.

### 3D Reconstruction

Serial inner ear sections stained with Hematoxylin and Eosin from newborn *+/hb*, *hb/hb* and *hb/Hmx3^KO^* mice were captured using a HV C20 CCD camera and imported into a frame grabber programme. Areas of interest on each section were then selected using a customised version of Image Pro. These selected areas were saved as tif files and Spyglass Slicer then rendered these as a solid 3D object.

### Transmission electron microscopy

Inner ears from fourteen-month-old *hb/hb* and *+/hb* littermates were fixed in glutaraldehyde, decalcified in 4%EDTA, post-fixed in 1% osmium tetroxide and saturated in uranyl acetate. Then the samples were dehydrated through an ethanol series and embedded in propylene oxide and Araldite resin (1∶1) and 3∶1 resin for 30 min and Araldite resin overnight under vacuum. The samples were embedded in fresh Araldite resin at 60 degree for 24–48 hours. Thin sections were taken from selected regions and examined using a Philips EM300 or 410 transmission electron microscope [25].

### *In situ* hybridisation and immunohistochemistry

For the marker analysis on sections, 3 embryos of each genotype were used and for whole mount *in situ* hybrisation 4 embryos of each genotype were used. For whole mount *in situ* hybridisation, embryos were fixed overnight at 4°C in 4% paraformaldehyde in PBS and processed as described [26]. For *in situ* hybridisation and immunohistochemistry on sections, samples were fixed overnight at 4°C in 10% neutral-buffered formalin, embedded in paraffin and cut into 8 µm sections and the Ventana Discovery system (Ventana Medical Systems, Inc Illkirch, France) was used according to the manufacturer's instructions. Plasmids containing cDNA of Bmp4 (Jones et al., 1991), and antibodies against Sox2 (Abcam, Cambridge, UK, cat. no. ab15830), Calretinin (Chemicon international, Millipore, Hampshire, UK, cat. no. AB5054), Myo7A (Proteus Bioscience, Ramona, CA, cat.no. 25-6790), P27^kip1^ (Cell Signaling Technology, Danversm cat. No. 711-065-152), Trp2 (Abcam, Cambridge, uk, cat.no ab74073), Laminin (Abcam, Cambridge, UK, cat.no ab11575), c-kit (Abcam, Cambridge, UK, cat. no. ab5506), Kcnj10 (Alomone Labs, Jerusalem, Israel, cat.no. APC-035), Hmx2 (Chemicon international, Millipore, Hampshire, UK, cat. no. AB5746), Hmx3 (Chemicon international, Millipore, Hampshire, UK, cat. no. AB5744), Nkx1.2 (Abcam, Cambridge, UK ab105940), Fgfr2 (Abcam, Cambridge, UK, cat.no ab10648), Chst15 (LifeSpan BioSciences, Seattle, WA, US, cat.no.LS-C82503/31418), Gpr26 (Abcam, Cambridge, UK, cat.no ab101606), Cpxm2 (Sigma-Aldrich. St.Louis, MO, US, cat.no. WH0119587M1) and Mast Cell Tryptase (Abcam, Cambridge, UK, cat.no ab2378) were used. Secondary antibodies were Jackson ImmunoResearch (West Grove, PA, USA) biotin conjugated donkey anti-rabbit (711-065-152), Jackson ImmunoResearch biotin conjugated donkey anti-goat (705-065-147) and Epitomics (Burlingame, CA, USA) anti-mouse IgG (3021-1).

Probes for the murine *Hmx2* (ENSMUSG00000050100) and *Hmx3* (ENSMUSG00000040148) genes were obtained by PCR on cDNA obtained from C57BL/6 embryos at E14.5 using specific primers designed with 3′ T7 promoter and 5′ T3 promoter tails. PCR primers were designed with the help of Primer3 (http://frodo.wi.mit.edu/primer3/input.htm. Accessed 2012 Aug 2.) and ordered from Sigma-Aldrich. They amplify a 200 bp conserved region which is upstream of the homeobox region (within the second exon) in both genes.

FHmx3_T3: 5′ AATTAACCCTCACTAAAGGGAG-CCTCTGCTCAAGGCTGACC-3′

RHmx3_T7: 5′ AATACGACTCACTATAGGGAG-GGCTTCTTCTCGGGACTCTC-3′

FHmx2_T3: 5′- AATTAACCCTCACTAAAGGGAGACGCCATTCCTTTCTCCTTC-3′

RHmx2_T7: 5′ AATACGACTCACTATAGGGAG-GACTCGAGCTGGTACACTTGG-3′

Digoxygenin-labelled RNA probes were generated by *in vitro* transcription using T3 and T7 RNA polymerases as described in [26].

Slides were photographed using a microscope with Nomarski optics (Axioplan; Zeiss) and digital camera (AxioCam; Zeiss). Images were acquired by the AxioCam MRc camera (Carl Zeiss), using the Axiovision 3.0 software (Carl Zeiss), with a format of 1030 _ 1300 pixels. Pictures were processed with Adobe Photoshop CS2.

### Whole mount immunofluorescence

Three pairs of littermates at postnatal day 4 (three *+/hb* controls, and three *hb/hb*, from three different litters), were fixed by immersion in 4% paraformaldehyde in phosphate buffered saline (PBS; pH 7.4) for 2 h at room temperature. Sensory tissue was dissected in PBS, permeabilized with 0.5% Triton X-100 for 30 min and blocked overnight at 4°C with 4% bovine serum albumin in PBS. Tissues were then incubated with primary antibody anti Chst15 (Sigma Aldrich, UK, cat. num. HPA017584, 1∶300 dilution) for 2 h, rinsed with PBS, stained with Alexa Fluor 488-conjugated anti-rabbit secondary antibody (Molecular Probes) for 1 h, counterstained with Alexa Fluor 594 phalloidin (0.001 U µl^−1^; Molecular Probes) and mounted using Prolong Antifade (Molecular Probes). Fluorescence confocal images were obtained with a Zeiss LSM 510 confocal microscope with a 63×1.4 numerical aperture objective.

### RNA extraction and quantitative RT-PCR

The heads of E12.5 embryos and the inner ears of P5 pups were dissected and stored at −20°C in RNAlater stabilization reagent (QIAgen, cat. no. 76106). RNA was extracted using QIAshredder columns (QIAgen, cat. no. 79654) and the RNeasy mini kit (QIAgen, cat. no. 74104), following the manufacturer's instructions. Quantitative RT-PCR was carried out on cDNA from the above tissue, using TaqMan probes and reagents from Applied Biosystems as already described in [27]. Statistical analysis was performed using one-way ANOVA followed by the Student's t test.

### Western blot analysis

Protein levels from 2 inner ears pooled together for each genotype were analyzed by Western blot as follows. Inner ear samples from each mouse at postnatal day five were homogenized using micropestles (Eppendorf AG, Hamburg, Germany) and lysed on ice in 400 µl ice-cold 50 nmol/L Tris.HCl, 5 mmol/L EDTA, 5 mmol/L EGTA, pH 7.5, 0.5% NP-40, with Roche Complete Mini protein inhibitors (cat.no. 11836153001). Cellular debris was removed by centrifugation at 16,000 rpm for 20 min at 4°C. Insoluble material was removed from the protein extracts by centrifugation at 13,000 rpm for 15 min at 4°C. The protein content in supernatant fractions was quantified (Nanodrop ND-8000, A280 assay) and aliquots were frozen in liquid nitrogen and stored at −80°C until use. 200 µg of inner ear protein extracts from *hb/hb* and control littermates were subjected to 10% SDS-PAGE. Western blots were probed with anti-mast cell tryptase antibody. GAPDH was used as loading control. Protein blots were probed for 2–4 h at room temperature with specific antibodies, followed by horseradish peroxidase-coupled secondary antibody, and analyzed using an ECL chemiluminescence system (Thermo Fisher Scientific UK, cat.no.34077). The antibodies (Abs) used were Mast Cell Tryptase (Abcam, Cambridge, UK, cat.no ab2378; 1∶1000) and Gapdh (Abcam, Cambridge, UK, cat.no ab9482; 1∶5000).

### Array CGH analysis using Agilent mouse genome 244A CGH microarrays

4 *hb/hb* mutants and 4 *+/+* controls aged from P1 and P30 were used for the aCGH. Genomic DNA was labeled using the BioPrime DNA Labeling Kit (Invitrogen, 18094-011). 450 ng of each experimental sample and reference were mixed with 60 µl of 2.5X random primer solution (Invitrogen) and nuclease-free water (Ambion, AM9937) to a final volume of 130.5 µl. Samples were denatured at 100°C for 10 min before being immediately cooled on ice. After addition of 15 µl of 10X dNTP mix (2 mM dATP, 2 mM dGTP, 2 mM dTTP, 1 mM dCTP), 1.5 µl of 1 mM Cy5-dCTP (experimental sample) or Cy3-dCTP (reference sample) (GE Healthcare, PA55321) and 3.0 ul of Klenow Fragment (40 U/µl) (Invitrogen), samples were incubated at 37°C for 16 h (overnight). Samples were purified using the PureLink PCR Purification Kit following the manufacturer's protocol (Invitrogen, K3100-01). Labeling efficiency was assessed using the Nanodrop-8000 Spectrophotometer (Thermo Scientific). Samples were hybridised to Agilent 244A mouse genome arrays following the manufacturer's protocol (G4410-90010). Microarrays were scanned using the Agilent DNA High Resolution Microarray Scanner (Agilent, G2505C) following the manufacturer's protocol. Raw image data was processed using Agilent's Feature Extraction software (v10.7.3.1). Bioconductor packages limma, DNAcopy and CNTools were used to analyse copy number variations [28]. Additionally, plots focusing on these regions were produced for visual confirmation using the “whole” option of “R”.

### Whole genome sequencing and sequence analysis of the headbobber mutation

Two *hb/hb* were used for whole genome sequencing. Sequencing was performed using Illumina Genome Analyzer II platform as paired-ends 100-bp reads according to the manufacturer's protocol. Four lanes of sequence were generated for each mouse. Sequences were mapped to the reference genome (NCBIm37) using bwa0.5.9 [29] and were improved by local realignment around insertions and deletions discovered in the mouse genomes project [30] using GATK 1.1–5 [31]. The lanes were merged and library duplicate fragments were marked using picard (http://picard.sourceforge.net, version 1.47. Accessed 2012 Aug 2.) and the merged BAM files were analysed using the Integrative Genomics Viewer (IGV) software [32]. Sequence data have been deposited in the European Nucleotide Archive (accession number: ERP000977).

## Results

### The headbobber inner ear phenotype

The mouse mutant headbobber (hb, MGI:2447989) harbours a recessive mutation that causes deafness, and vestibular dysfunction that is manifested as circling, hyperactivity and head bobbing. The headbobber mutation arose by transgenic insertion in a line carrying a plasmid phβAPr-1neo, which includes a portion of the human beta actin promoter [4]. Only homozygotes are affected suggesting recessive inactivation of one or more genes. *hb/hb* showed abnormal response to the air righting, contact righting, Elevated Platform, Negative Geotaxis and reaching and swimming behavioural tests (data not shown). Moreover, they do not show any Preyer reflex in response to sound.

**Figure 1:**
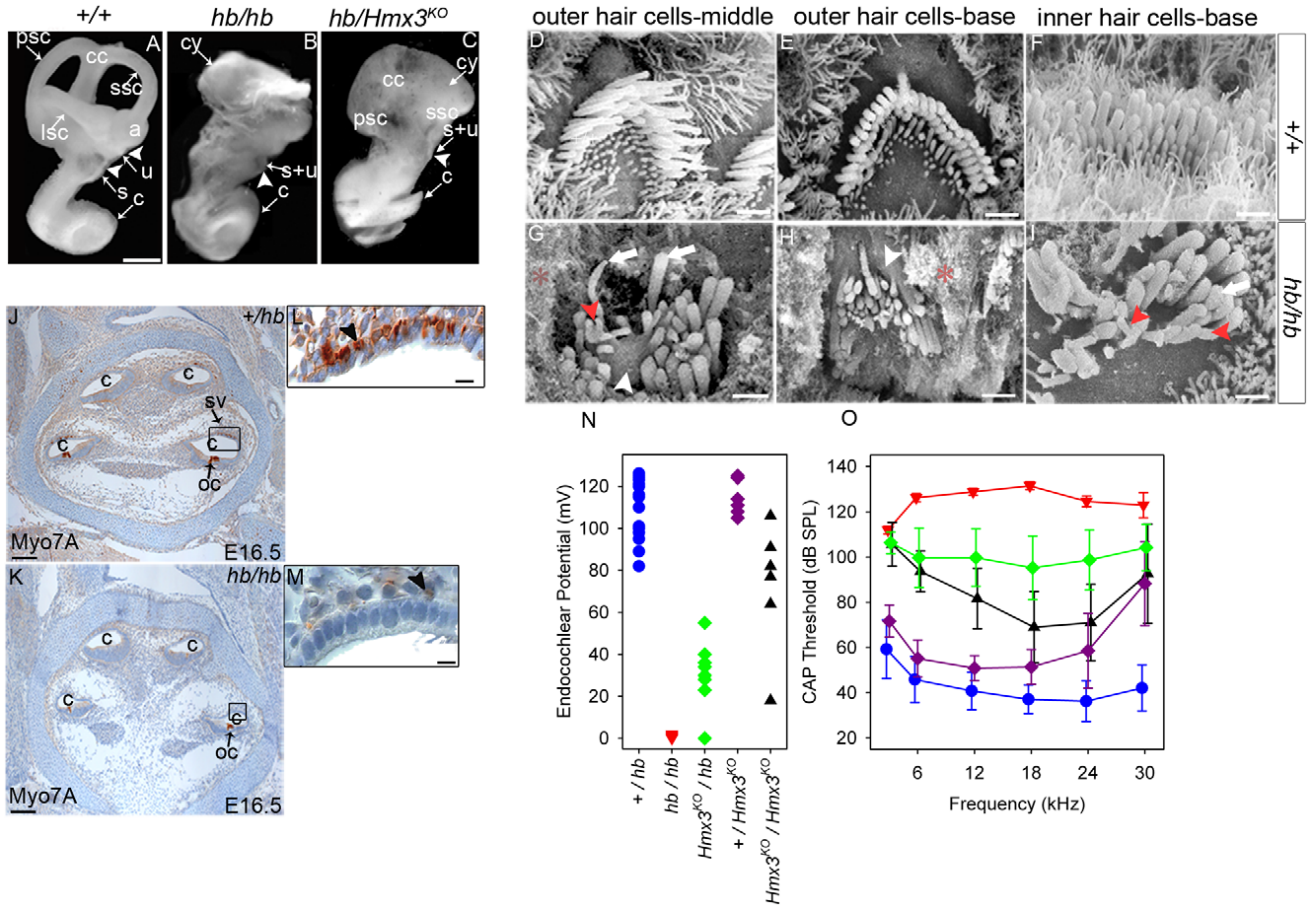
Overview of the headbobber inner ear phenotype. **A–C**: Headbobber inner ear gross morphology analysed by paintfilling of newborn *hb/hb, hb/Hmx3^KO^* and control littermates, clearly showing the gross malformation of the semicircular canals (cy) observed in *hb/hb* and *hb/Hmx3^KO^*. Scale bar: 0.5 mm. **D–I:** SEM of organ of Corti of *hb/hb* and control littermates at P5. In the basal and middle turns of the cochlea, hair bundles in *hb/hb* look severely disorganized if compared with the one displayed by the littermate controls, showing bifurcations (red arrowheads in G and I) and fusion of stereocilia (white arrowheads in G and H). Height and thickness of stereocilia are affected too in *hb/hb* mutants (white arrows). *: debris from collapse of Reissner's membrane. Scale bar:1.5 µm. **J–M**: Myo7A expression in *hb/hb* mutants and control littermates at E16.5. Myo7A marks melanoblasts in stria vascularis and we did not detect any melanoblasts in the *hb/hb* developing stria vascularis when compared to littermate controls (arrowheads in L,M). Scale bars: J,K: 200 µm; L,M: 5 µm. **N–O:** Endocochlear Potential (N) and Compound Action Potential (O) measurements on a total of fifteen *hb/hb* (red triangles), seventeen *+/hb* (blue circles), six *Hmx3^KO^/Hmx3^KO^* (black triangles), eight *Hmx^KO^/hb* (green diamonds) and and eight *+/Hmx3^KO^* (purple diamonds), all aged 60 to 102 days. Individual EP measurements are plotted in N, and means +/− SD are plotted for compound action potential thresholds except for *hb/hb* (red triangles) where there were no responses, so the maximum sound level used is plotted (if *hb/hb* had any response, thresholds would be above this level). a: ampulla, bc: basal cell, bv: blood vessel, c:cochlea, cc:common crus, cy: vestibular cyst, ed: endolymphatic duct, ic: intermediate cell, int: interdigitation, lsc:lateral semicircular canal, mc: marginal cell, oc:organ of Corti, psc: posterior semicircular canal, rm: Reissner's membrane, s: saccule, ssc: superior semicircular canal, s+u: utriculosaccular space, sv: stria vascularis, u: utricle.

Consistent with its early onset strong vestibular phenotype, *hb/hb* mutants display a gross vestibular dysmorphology (Fig. 1B). *hb/hb* mutants show a cyst-like vestibular system, with no clear evidence of semicircular canals. 3D reconstruction of paraffin wax inner ear serial sections from newborns show the presence of an abnormal endolymphatic duct in *hb/hb* (Figure S2 G–H). The cochlea, however, has a normal gross morphology compared to littermate controls (Fig. 1 A–B).

*hb/hb* mice are profoundly deaf as demonstrated by the absence of any compound action potential responses (Fig. 1O). Endocochlear potentials are around zero, compared to the normal levels of 80–120 mV (Fig. 1N). Scanning electron microscopy of the organ of Corti revealed that in *hb/hb*, both inner and outer hair bundles look severely disorganized and are arranged in randomly positioned clumps rather than in the normal‘V’-shaped pattern (Fig. 1D). In detail, hair cells in the basal and middle turn contain elongated stereocilia often fused at the stereocilia base (white arrowheads in Fig. 1 G,H) and showing bifurcations (red arrowheads in Fig. 1G,I). Stereocilia polarity, thickness and height seem to be affected in *hb/hb* when compared to their littermate controls (arrows in Fig. 1 G,I), with membranes looking in a state of collapse in mutants. (* in Fig. 1G,H).

We have used Myo7A to detect the presence of neural crest-derived melanoblasts (precursors of intermediate cells) in developing stria at E16.5, and we do not detect melanoblasts in the *hb/hb* developing stria compared to littermate controls (Fig. 1J–M). Later in development, as shown in Figure S3, the stria vascularis looks severely disorganized in *hb/hb* adult mutants compared to littermate controls, lacking the usual extensive cellular interdigitation. No intermediate cells are detected in *hb/hb* stria and Reissner's membrane is collapsed.

Functional and anatomical studies of the mouse and human inner ear have identified the same broad categories of pathology in the two species: morphogenetic, cochleo-saccular and neuroepithelial [33]. Morphogenetic abnormalities involve gross structural deformities of the labyrinth. A strial abnormality is the primary cochleo-saccular defect in which there is a reduced or absent endocochlear potential (EP) and sometimes collapse of Reissner's membrane, which eventually results in degeneration of the organ of Corti. Neuroepithelial defects originate in the organ of Corti and do not affect the stria vascularis directly [34]. Taken together, our results allow us to consider headbobber as a combined morphogenetic and cochleo-saccular mutant and identification of the genes affected by the mutation will provide important information on the biological basis of normal hearing and vestibular system development.

### The headbobber mutation

The headbobber mutation and the transgenic insertion have been independently mapped to the same region of chromosome 7, 65 cM from the centromere between the markers *D7Mit105* and *D7Mit12*, using two separate backcrosses (Figure S1 AB). The headbobber region is 8 Mb and contains 86 annotated genes.

**Figure 2:**
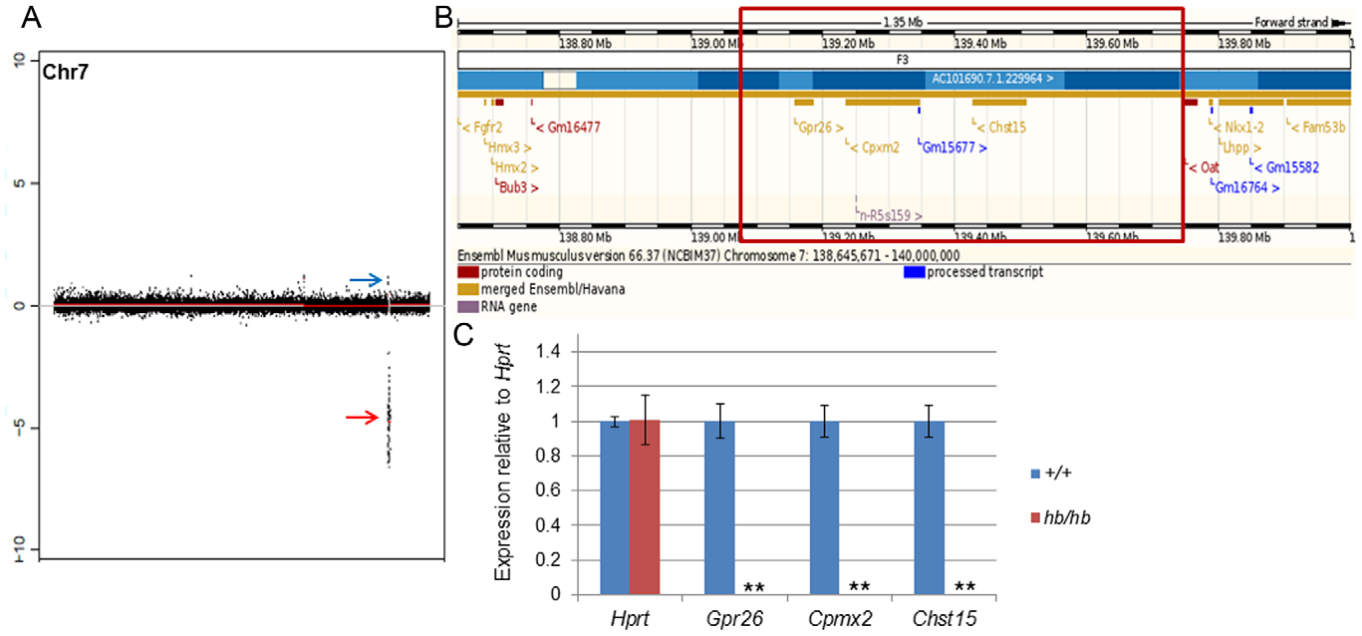
The headbobber mutation. **A:** Plot showing the main genomic variations found by aCGH in 4 *hb/hb* on Chromosome 7: a 647.7 kb deletion in qF3, confirmed by deep sequencing (red arrow, loss of copy number, Chr7: 139061190–139708657, log ratio of −8.5) and a gain in qF3 (blue arrow, Chr7: 138832773–138832773, log ratio 1.3). **B:** Screenshot of the deleted region (www.ensembl.org). The red square delimits the breakpoints of the deletion. There are three protein coding genes in the region (*Gpr26*, *Cpxm2* and *Chst15*), a non-coding RNA (*n-R5s159*) and an antisense RNA (*Gm15677*). **C**: Quantitative real-time PCR on cDNA generated from RNA from P1 cochleae show the absence of mRNA for *Gpr26*, *Cpxm2* and *Chst15* in *hb/hb* compared to the littermate controls. Error bars, s.d. Quantity normalised to *Hprt1* levels. N = 3.**: p<0.01.

In order to narrow down the headbobber region and define the physical effect of the transgenic insertion on Chr7, we performed array comparative genomic hybridization (aCGH) on four *hb/hb* mice to compare to wild type controls as well as whole genome sequencing on two mutants. In detail, aCGH is a highly useful tool to define DNA copy number variations (either deletions or duplications), and therefore identify zones of aberrations in the genome [28]. Consistent with the results we got from the genetic mapping, our aCGH and deep sequencing data detect a ∼ 648 kb deletion on Chr7 in all the mutant samples (Chr7: 139061190–139708657; Fig. 2A,B), which maps 375 kb telomeric to the *Hmx3* locus and 78 kb centromeric to *Nkx1.2*. We also detected an insertion from mouse chromosome X at the end of the deletion (Chr7:139708658–139709168). The inserted sequences correspond to the genomic region ChrX: 159137545–159137781 and no genes are annotated in this region.

**Figure 3:**
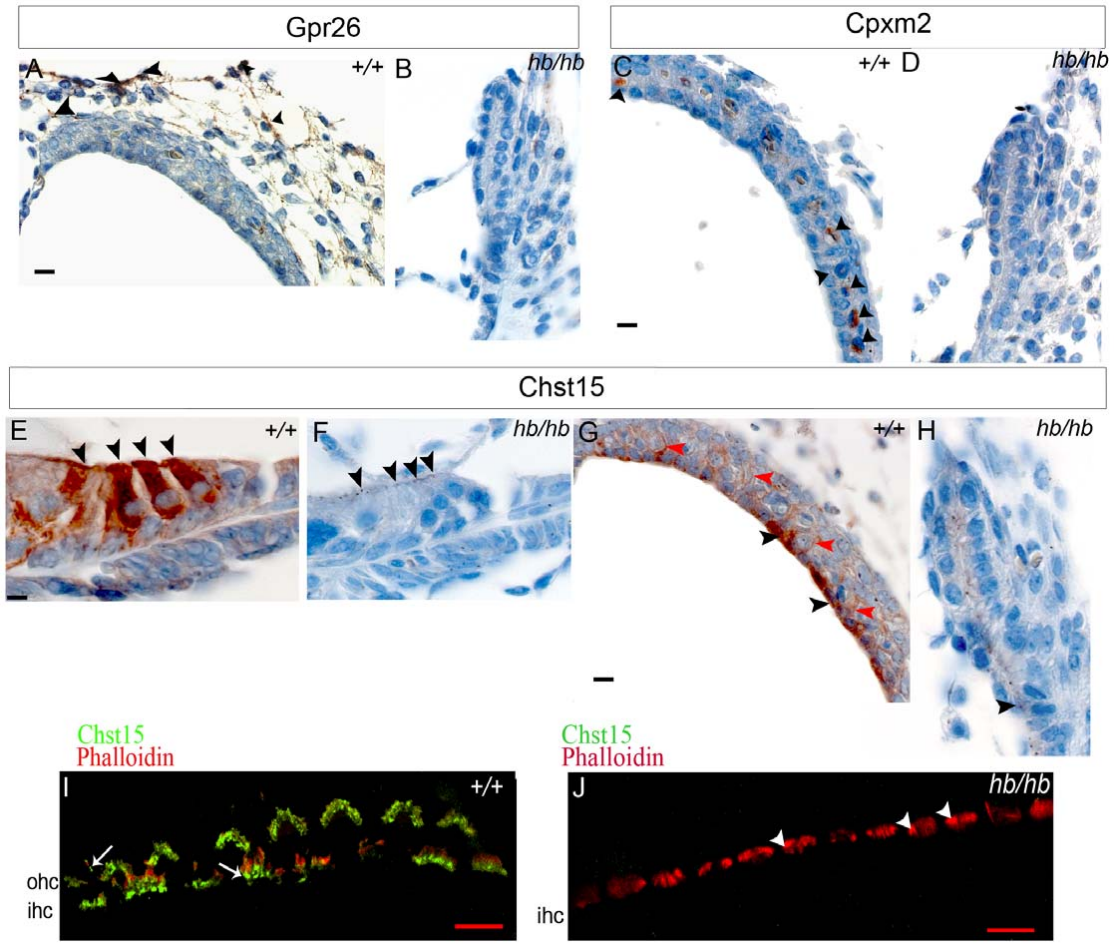
Cochlear expression of genes in the headbobber deletion on Chr7. **A–B:** Immunohistochemistry on ear sections at postnatal day five shows Gpr26 expression in spiral ligament fibrocytes (black arrowheads in A). We did not detect presence of Gpr26 in *hb/hb* cochleae at this stage (B). Scale bar: 10 µm. **C–D:** Immunohistochemistry on ear sections at postnatal day five shows Cpmx2 expression specifically in intermediate cells of stria vascularis (arrowheads in C). No protein was detected in *hb/hb* (B). Scale bars: 10 µm. **E–H:** Immunohistochemistry for Chst15 in *hb/hb* and control littermates at P5. At this stage, Chst15 shows expression in hair cells (arrowheads in E), marginal cells (black arrowheads in G) and their interdigitation to intermediate cells of stria vascularis (red arrowheads in G). As shown in F and H, we cannot detect any significant expression of Chst15 in *hb/hb* compared to littermate controls in the organ of Corti (F) and stria vascularis (H), although we can still detect a few protein dots (black arrowheads in F,H), which can either be an artifact or due to non-specific staining. Scale bar: 10 µm. **I–J:** Whole mount immunofluorescence of organ of Corti at postnatal day five shows expression of Chst15 (green) in the basal region of stereocilia of both inner and outer hair cells (arrows in I). We do not detect any Chst15 expression in the *hb/hb* disorganized hair bundles (arrowheads in J). Phalloidin (red) stains actin filaments of stereocilia. Scale bars: 10 µm. ohc: outer hair cells; ihc: inner hair cells.

There are three protein coding genes annotated in the deleted region on Chromosome 7: G protein-coupled receptor 26 (*Gpr26*), carboxypeptidase X 2 (*Cpxm2*) and carbohydrate (N-acetylgalactosamine 4-sulfate 6-O) sulfotransferase 15 (*Chst15*) (Fig. 2B). Here we report their expression in the mouse inner ear at early postnatal days (Fig. 3), which suggests their deletion can contribute to the headbobber hearing phenotype.

***Gpr26*** (NM_173410.3) is an Orphan G-coupled receptor [35], [36]. It displays a significant level of constitutive activity and it is widely expressed in selected tissues of human brain as well as the developing and adult mouse brain [37]. *Gpr26* deficient mice have been reported to show anxiety and depression-like behaviour [38]. We detect absence of *Gpr26* expression in *hb/hb* compared to littermate controls throughout the whole cochlea (Fig. 3A,B). In wildtype mice at postnatal day five, *Gpr26* expression is detected in the spiral ligament fibrocytes (Fig. 3A), in vestibular system fibrocytes and spiral ganglion (data not shown).

***Cpxm2*** (NM_018867.5) is transcribed from the opposite strand to *Gpr26*, so that they are actually back to back on Chr7. *Cpxm2* has been proposed to be a binding protein with a role in cell adhesion, more than an enzyme [39]. It is reported to be expressed in mouse brain [39] as well as developing cochlea [40]. Our expression analysis shows *Cpxm2* expression confined in intermediate cells of stria vascularis at P5 (Fig. 3C) and no expression is detected in *hb/hb* compared to littermate controls (Fig. 3D).

***Chst15*** (NM_029935.5) shows various physiological activities through interacting with numerous functional proteins [41] It is transcribed in the same direction as *Gpr26* and it is reported to be expressed in developing brain [40], [42], bone marrow-derived mast cells [43] and early mouse embryos [41]. Mice deficient in *Chst15* have not been tested for hearing impairment; however, their hematopoietic phenotype has been analysed and they exhibit decreased protease activity in bone marrow-derived mast cells [43]. We detect expression of Chst15 in the apical region of both inner and outer hair cells (arrowheads in Fig. 3E), as well as in their cell bodies. Consistent with the headbobber strial phenotype, we detect Chst15 expression in marginal cells and their interdigitations (black and red arrowheads respectively in Fig. 3G) in stria vascularis. Furthermore, we have observed its expression in the base of stereocilia by whole mount immunofluorescence (arrows in Fig. 3I), which could explain the stereocilia phenotype we observe in *hb/hb*, with extensive fusion at their base (Fig. 1G). As expected, we observe a complete loss of Chst15 in stereocilia of *hb/hb* compared to littermate controls (Fig. 3J). Our expression analysis showed absence of significant Chst15 expression in *hb/hb* organ of Corti and stria vascularis (Fig. 3F,H) compared to littermate controls.

Quantitative RTPCR on *hb/hb* cochleae from mice aged P1 confirmed absence of transcription for the three genes included in the deletion (Fig. 2C).

### *Hmx3*, *Hmx2* and *Nkx1.2* homeobox transcription factors are downregulated in *hb/hb*

**Figure 4:**
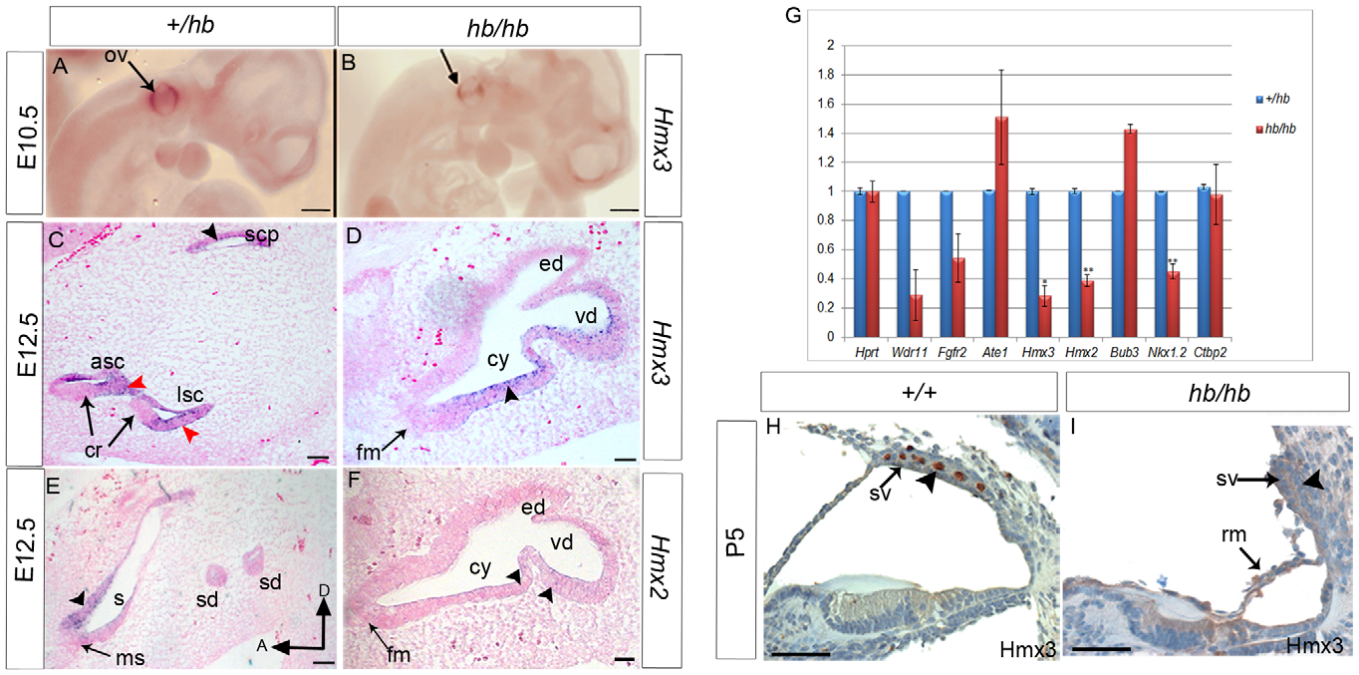
Expression analysis of *Hmx3* and *Hmx2* homeobox transcription factors in *hb/hb* and control littermates. **A–B:** *Hmx3* expression in *hb/hb* mutants tested by whole mount RNA *in situ* hybridisation at E10.5, showing decreased expression of *Hmx3* in the dorsal part of the otocyst of *hb/hb* mutants compared to littermate controls (arrows) **C,D:** RNA *in situ* hybridisation for *Hmx3* at E12.5 in vestibular system sagittal sections. In control littermates, *Hmx3* RNA is detected in the canal fusion plate (black arrowhead in C), semicircular canals (red arrowheads in C), and in the utricle and saccule (not shown) but its expression is always observed in non-sensory epithelial cells as previously reported (C). In *hb/hb* mutants we still detect *Hmx3* mRNA in the vestibular non-sensory regions compared to the littermate controls (arrowhead in D). **E,F:** *Hmx2* expression in vestibular system detected by *in situ* hybridisation on sagittal sections from *hb/hb* and littermate controls at E12.5. In control mice, as previously reported, *Hmx2* shows a similar expression pattern to *Hmx3* in the non-sensory cells and in the canal plate, in the utricle (arrow in *E*) and in the canals (not shown). In *hb/hb* we detect *Hmx2* expression only in a few cells in the non-sensory regions of the structurally abnormal vestibular system, compared to the littermate controls (arrowheads in *F*). Scale bars: A,B, 0.5 mm; C–F, 100 µm. **G:** Quantitative real-time PCR of cDNA generated from RNA from E12.5 littermate embryo half heads. Only *Hmx3, Hmx2* and *Nkx1.2* mRNA levels are significantly downregulated in *hb/hb* compared to littermate controls. Error bars, s.d. Quantity normalised to *Hprt1* levels. N = 3. *:p<0.05; **: p<0.01. **H–I:** Expression of Hmx3 in *hb/hb* cochlear sections and control littermates at postnatal day five. After birth, Hmx3 is expressed in intermediate cells in stria vascularis (arrowhead in H, see also Figure S2). As it is clear in I, no Hmx3 staining is detected in *hb/hb* cochlea at P5, consistent with the loss of intermediate cells (arrowhead in I). Scale bars: 20 µm. a: anterior, asc: anterior semicircular canal, cr:cristae, cy: vestibular cyst, D: distal; ed: endolymphatic duct fm: fused maculae lsc: lateral semicircular canal mu: maculae utriculi ms: maculae sacculi; oc: organ of corti, ov: otic vescicle; rm: Reissner's membrane, s: saccule scp: superior canal plate; sd: semicircular duct; s+u: utriculosaccular space, sv: stria vascularis, vd: vestibular diverticulum.

Among numerous candidate genes for the headbobber mutation which map in the *hb* region, our attention was focused on homeobox transcription factors *Hmx3* (*Nkx5.1*, NM_008257.3) and *Hmx2* (*Nkx5.2*, M_145998.3), as single and double knockout mice show a severe malformation of the vestibular region of the inner ear [10], [19], [44]–[46], similar to what we observe in the *hb/hb* mouse. Therefore, a complementation test was carried out between headbobber homozygotes and the *Hmx3 ^KO^* mice [20]. We crossed +/*Hmx3^KO^* to *hb/hb* mice and observed that around half the offspring (those carrying one *hb* allele and one genotyped *Hmx3^KO^*) displayed a circling phenotype indicating non-complementation. Newborn compound heterozygotes were paintfilled and showed a severely affected inner ear structure. These mice have a fused saccular and utricular region and very rudimental posterior and superior semicircular canal. The cochlea develops normally, and looks similar to that of *hb/hb* mutants (Fig. 1C). The endolymphatic duct looks normal in *hb/Hmx3^KO^* compound mutants, excluding an essential role for *Hmx3* in endolymphatic duct development, as already reported [44] (Figure S2). We measured compound action potential responses and endocochlear potentials in *Hmx3^KO^/Hmx3^KO^, Hmx3^KO^/hb* and littermate controls and detected a wide range of intermediate values in *Hmx3^KO^* homozygotes compared to *hb/hb* and littermate controls, while compound heterozygous *Hmx3^KO^/hb* show intermediate responses compared to *hb/hb* and *Hmx3^KO^* (Fig. 1N,O). These data suggest genetic interaction between *Hmx3* and the genes affected by the headbobber mutation leading to multigenic inheritance of the inner ear phenotype. We sequenced the coding regions of *Hmx3* and *Hmx2* in the *hb/hb* mutants by Sanger sequencing and we did not detect any changes in the coding structure (data not shown). We then decided to analyse their expression levels in the headbobber inner ear at different stages of development, in order to understand whether the transgenic insertion associated with the mutation has any effect on their expression. *Hmx3* is considered one of the earliest markers of inner ear development, as its expression is detected as early as E8.5 in the dorsolateral part of the otocyst [47]. We detect reduced labeling by *in situ* hybridisation of *Hmx3* in the dorsal part of otocyst at E10.5 in *hb/hb* mutants compared with littermate controls (Fig. 4A,B). *Hmx3* mRNA labelling is also reduced in *hb/hb* mutants compared to littermate controls at E12.5, suggesting again its involvement in the headbobber phenotype (Fig. 4C,D). At E12.5, *Hmx3* expression is detected in the canal fusion plate, in the semicircular canals and in some cells in the utricle and in the saccule in control mice; its expression is restricted to non-sensory epithelial cells. Only a few non-sensory cells express detectable *Hmx3* in the *hb/hb* vestibular structure (Fig. 4D) and the positive region could correspond to the fusion plate. As expected, *Hmx3* is not expressed in the cochlea at this stage (data not shown). Reduced labelling for *Hmx3* is detected by *in situ* hybridization in *hb/hb* brain compared to littermate controls (data not shown). As *Hmx2* starts to be expressed only several hours after *Hmx3* [46] we analysed its mRNA levels in the headbobber inner ear at E12.5 and, as expected, *Hmx2* shows an overlapping spatial pattern of expression with *Hmx3* at this stage, with expression in the epithelia of the canal plate, semicircular canals and in the utricule and saccule (Fig. 4E). In *hb/hb*, we detect reduced mRNA levels for *Hmx2* in the vestibular system at this stage when compared to littermate controls, with only a few *Hmx2* positive cells detected (Fig. 4F). As expected, *Hmx2* is not expressed in the cochlea at this stage (data not shown). Moreover, we analysed the Hmx3 and Hmx2 protein levels in the inner ear by immunohistochemistry at postnatal day 5 in control mice, and we detect their expression in intermediate cells, as previously reported [47]. However, we do not detect any staining for Hmx3 and Hmx2 in *hb/hb* mutants. (Fig. 4 H–I, Figure S2A–B and data not shown).

**Figure 5:**
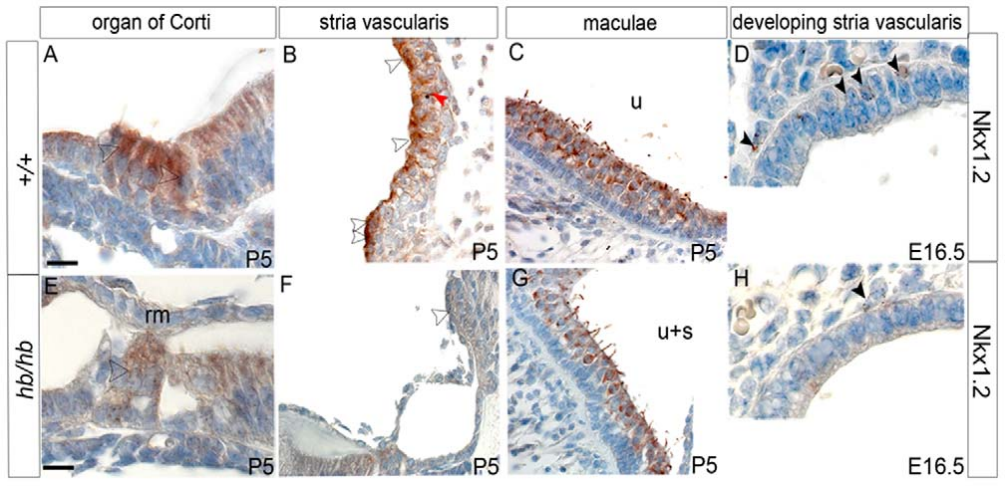
Expression pattern of Nkx1.2 in *hb/hb* and control littermates at postnatal day 5. Nkx1.2 shows expression in hair cells of organ of Corti (arrowhead in A), marginal cells and marginal cells processes in stria vascularis (white and red arrowheads in B, respectively) and hair cells in maculae (C) at P5. Moreover, Nkx1.2 is detected in the perinuclear area of melanoblasts in developing stria vascularis (arrowheads in D). Nkx1.2 staining is reduced in *hb/hb* compared to littermate controls (arrowheads in E–H). Scale bars: A,B,C,D,E,G,H: 10 µm; F: 20 µm.

Quantitative RT-PCR confirms that *Hmx2* and *Hmx3* are significantly downregulated in mutant embryonic heads compared with controls (Fig. 4G), together with *Nkx1.2* (NM_009123.2), another homeobox transcription factor (from the NK-1 class of homeobox genes) mapping in the headbobber locus in a reverse transcriptional direction to *Hmx2* and *Hmx3*. *Nkx1.2* has been reported as transiently expressed in the developing posterior CNS [48], but nothing is known of its expression in the inner ear and putative function in hearing. We sequenced its coding regions and did not find any mutation in *hb/hb* DNA (data not shown). Our expression analysis at postnatal day five shows that Nkx1.2 is expressed in inner and outer hair cells, marginal cells in the stria vascularis and sensory regions in the vestibular system (Fig. 5). Moreover, Nkx1.2 is expressed in prospective intermediate cells of the stria vascularis (melanoblasts) at E16.5 (Fig. 5D). Although we can detect nuclear staining (arrowheads in Fig. 5A,B), as expected, the transcription factor Nkx1.2 seems to be expressed in the cytoplasm as well, suggesting that the protein might need specific activation to translocate to the nucleus as has been observed for other transcription factors [49].The Nkx1.2 cochlear expression pattern clearly fits with the headbobber phenotype, suggesting a possible role for this homeobox transcription factor in melanoblast survival. Quantitative RTPCR confirms that Nkx1.2 expression in the inner ear is reduced at this stage compared to littermate controls (Fig. 5E,H).

We tested mRNA levels of other candidate genes in the headbobber region at E12.5, such as *Wdr11* (NM_172255.3), *Fgfr2* (NM_201601.2), *Ate1* (NM_166380.2) *Bub3* (NM_009774.3) and *Ctbp2* (NM_009980.4) by quantitative RT-PCR and found no significant differences in levels (Fig. 4G). In the case of *Fgfr2* [50] we also tested the inner ear expression pattern by immunohistochemistry at postnatal day five, but we did not detect any difference in *hb/hb* compared to littermate controls (Figure S2 E,F). These results suggest involvement of three homeobox transcription factors *Hmx3*, *Hmx2* and *Nkx1.2* in the headbobber phenotype, with the transgenic insertion not disrupting their coding sequences, but perhaps disrupting their (shared) regulatory elements on chromosome 7.Therefore, we used the TRANSFAC database [51] to map target binding sites (TFBSs) for 39 transcription factors, with a particular interest in those already reported to have a role in hearing and vestibular function [46], [52]–[54]. We discovered that *Hmx2, Hmx3* and *Nkx1.2* themselves, together with 10 other members of the *Nkx* gene family (table 1), are predicted to recognise high-affinity binding sites in the deleted region, suggesting a possible mechanism of transcriptional auto-regulation for these transcription factors. Moreover we found that a number of homeobox transcription factors have predicted binding sites in the deletion, including *Emx2*, *Hoxa2*, *Otx1*, *Otx2*, *Lmx1a*, *Lmx1b*, *Pax2*, *Pax6*, *Six1* and *Six2* (Table 1). Our analysis suggests that all these transcription factors might be involved in the regulation of *Hmx3*, *Hmx2* and *Nkx1.2* transcriptional levels.

**Table 1:** Transcription factors binding with high affinity in the region of the headbobber deletion on Chr7, identified with the use of the TRANSFAC database.

Transcription Factor	Number of high affinity binding sites	Described role in inner ear development
*Hoxa2*	1	+ [78]
*CREB*	3	-
*Pax2*	25	+ [79]
*Pax6*	2	-
*Six1*	2	+ [70], [80]
*Six2*	1	+ [81]
*Sp1*	2	-
*NF-X*	8	-
*Emx2*	1	+ [82]
*Oct-1*	4	-
*Lmx1a*	1	+
*Lmx1b*	2	+ [83]
*Otx1*	2	+ [84], [85]
*Otx2*	2	+ [84], [85]
*Nkx1.1*	1	-
*Nkx1.2*	1	In this work
*Nkx2.1*	2	-
*Nkx2.2*	1	-
*Nkx2.3*	2	-
*Nkx2.5*	1	-
*Nkx2.6*	2	-
*Nkx2.9*	1	-
*Nkx6.1*	2	-
*Nkx6.3*	4	-
*Hmx1*	2	-
*Hmx2*	1	+ [44]
*Hmx3*	1	+ [20]
*Lhx1*	1	+ [86]
*NF-y*	2	-
*c-Fos*	2	-

### Expression analysis of prosensory markers in the headbobber inner ear

The development of the inner ear is a very complicated process, which is characterized by a series of genetically programmed events. Although a good number of developmental regulators are known to be involved in this cascade of events, it is not yet clear what role these different genes play in development of the sensory patches, and in relation to one another [55]. In order to gain more insight on the role of the *Hmx* transcription factors *Hmx3*, *Hmx2* and *Nkx1.2* in this pathway, we have performed an expression analysis of molecular markers of key events in development on headbobber ear sections at different stages of development. This has been performed either by RNA *in situ* hybridisation or by immunohistochemistry.

**Figure 6:**
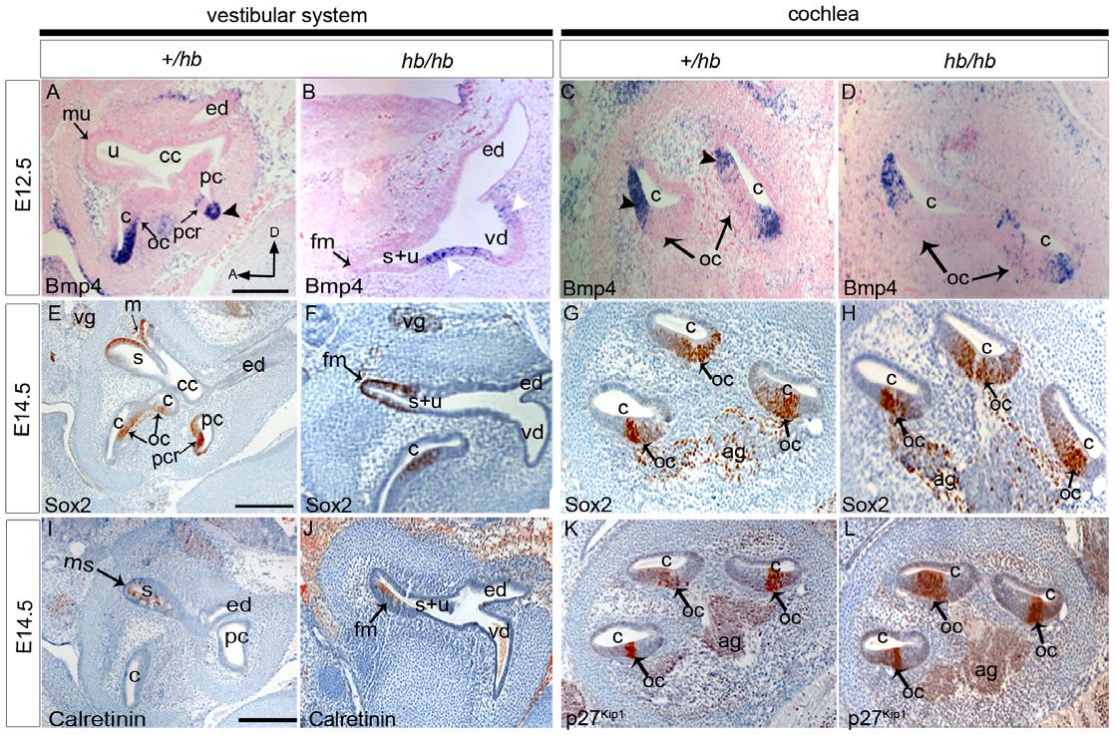
Summary of the expression patterns of selected markers of inner ear development, performed on sagittal sections from *hb/hb* mutants and littermate controls at different stages of development. **A–D:** RNA *In situ* hybridisation for *Bmp4* E12.5 in *hb/hb* and littermate controls. In control mice, *Bmp4* expression overlaps with the pattern detected for *Hmx3* and *Hmx2*, being expressed in the non-sensory cells adjacent to the organ of Corti and cristae (arrowheads in A and C). No *Bmp4* expression is detected in maculae at that stage. (mu in A) In *hb/hb*, *Bmp4* is expressed in two regions in the vestibular cyst (white arrowheads in B), which are definitely not adjacent to any sensory regions in the headbobber homozygotes at later stages. C,D*:* No difference in *Bmp4* RNA levels has been detected in *hb/hb* mutant cochleae compared to their littermate controls. **E–H:** *Sox2* immunohistochemistry in *hb/hb* and littermate controls at E14.5. In control mice, *Sox2* is expressed in all the prosensory regions of the inner ear (arrows in E,G). In *hb/hb* vestibular system, *Sox2* shows normal expression in the fused maculae (fm in F), which is the only vestibular prosensory patch we detect in *hb/hb*. *Sox2* cochlear expression in *hb/hb* looks normal when compared with the littermate controls, suggesting a normal development of the organ of Corti at embryonic age E14.5. In addition, *Sox2* marks the nuclei of both vestibular and cochlear ganglia (vg in E,F and ag in G,H), and again no differences in *Sox2* expression have been detected in these cells at this stage of development. **I–J**: Expression of Calretinin at E14.5 of *hb/hb* and control littermates. At this stage Calretinin marks the developing hair cells. While the hair cells in the organ of Corti are not developing yet at this stage, a few hair cells start to develop in the maculae of normal mice (arrow in I). Calretinin expression analysis shows presence of a few normally developing hair cells in the fused maculae of *hb/hb* at this stage (arrow in J). **K–L:** p27^Kip1^ expression on *hb/hb* and littermate controls at E14.5. P27^Kip1^ at E14.5 is upregulated in cells of the sensory patch in the cochlea as they prepare to exit the cell cycle. Immunohistochemistry using P27^Kip1^ antibody demonstrates that in *hb/hb* mutants prosensory cells this marker is expressed in the same way in the *hb/hb* organ of Corti, compared to littermate controls (arrows in K,L). Scale bars: 200 µm A: anterior; ag: acoustic ganglion; c: cochlea; cc: common crus; cy: vestibular cyst;D: distal; ed: endolymphatic duct; fm: fused maculae; ms: maculae sacculi; mu: maculae utriculi; oc:organ of Corti; pc: posterior semicircular canal; pcr: posterior cristae; vg: vestibular ganglion; s:saccule; sv: stria vascularis; u:utricle; s+u: utriculosaccular space; vd: vestibular diverticulum.

The secreted factor *Bmp4* is expressed in the developing cristae and in the cochlea at E12.5 (Fig. 6A–D, [56]. In *hb/hb* mutants, *Bmp4* shows a comparable mRNA expression to littermate controls in the cochlea (Fig. 6C,D), with normal expression in the epithelium on the outer edge of the developing organ of Corti, confirming the normal establishment of this cochlear prosensory patch in the headbobber mouse model (Fig. 6D). As early as E12.5, as shown in Figure 6B, *hb/hb* already show an abnormal vestibular structure, with no presence of semicircular canals with their cristae but with formation of only one sensory patch in the utriculosaccular space (fused maculae, arrow in Fig. 6B). *Bmp4* mRNA is detected in two regions which, according to the expression screening performed at later stages, are definitely not going to develop into sensory regions in the *hb/hb* mutants (white arrowheads in Fig. 6B). Most likely, the two positive regions could be early developing cristae that as a result of the headbobber mutation, fail to specify as a mature sensory patch at later stages. In fact, a similar scenario was observed in *Hmx2* but not *Hmx3* knockout mice [44], [45], all these data suggesting that *Bmp4* might need full expression of *Hmx2* for its role in driving final cristae specification.

The developmental stage E14.5 is crucial for studying inner ear development, as at this stage prosensory regions start to be established and prosensory cells start to exit the cell cycle to differentiate and become mature sensory cells. *Sox2*, a member of the group B Sox (SRY-related HMG box) transcription factor family, is required for establishment of the prosensory regions in the inner ear [57]. It is expressed in all the prosensory regions and the developing neurons that will be innervating the sensory hair cells in control mice. In *hb/hb*, Sox2 shows expression in the fused maculae, organ of Corti and ganglia (Fig. 6F,H). No evidence of crista formation has been observed in any of the homozygotes labelled with Sox2 (Fig. 6F). At E14.5 Calretinin antiserum labels the postmitotic differentiating hair cells [58] in maculae. Immunohistochemistry with Calretinin antibody showed expression in some cells in maculae sacculi, cristae and vestibular ganglion in control mice (Fig. 6I and data not shown) and a few labelled cells were observed in the fused maculae in *hb/hb* mutants (Fig. 6J). While at E14.5 hair cell differentiation has already started in maculae and cristae, in the organ of Corti this process starts a little bit later in development, and therefore we do not detect any calretinin expression in cochlea at this stage either in *hb/hb* or in littermate controls (data not shown). p27^Kip1^ is a cyclin-dependent kinase inhibitor which is upregulated in cells of the sensory patch in the cochlea as they prepare to exit the cell cycle [59]. Again, p27^Kip1^ has a comparable expression in *hb/hb* mutants and their littermate controls (Fig. 6K,L). All these results demonstrate that *Hmx3, Hmx2* and *Nkx1.2* do not act in early specification and differentiation of hair cells once the location of the sensory patches has been determined.

### The headbobber stria vascularis remains immature with lack of intermediate cells and impaired basal lamina degradation

We investigated in detail the possibility that the lack of endocochlear potential in the *hb/hb* mutants could be directly related to a primary defect in stria vascularis, similar to what has been described for the melanocyte-deficient deaf mouse *viable dominant spotting* [25] and the *Varitint-waddler-J* deaf mutants [34]. In support of this hypothesis, we observed that the three genes deleted in headbobber show an intriguing expression in the stria vascularis after birth (Figure 3) as well as during development (data not shown).

To this purpose, we used an antibody to Myo7A [60] to detect the presence of neural crest-derived melanoblasts (precursors of intermediate cells) in the developing stria at E16.5, and we do not detect melanoblasts in the *hb/hb* developing stria compared to littermate controls (Fig. 1). However, we used Kcnq1 [61] as a marker for marginal cells and show that they are still present in the *hb/hb* abnormal stria vascularis (Figure S2 C,D).

**Figure 7:**
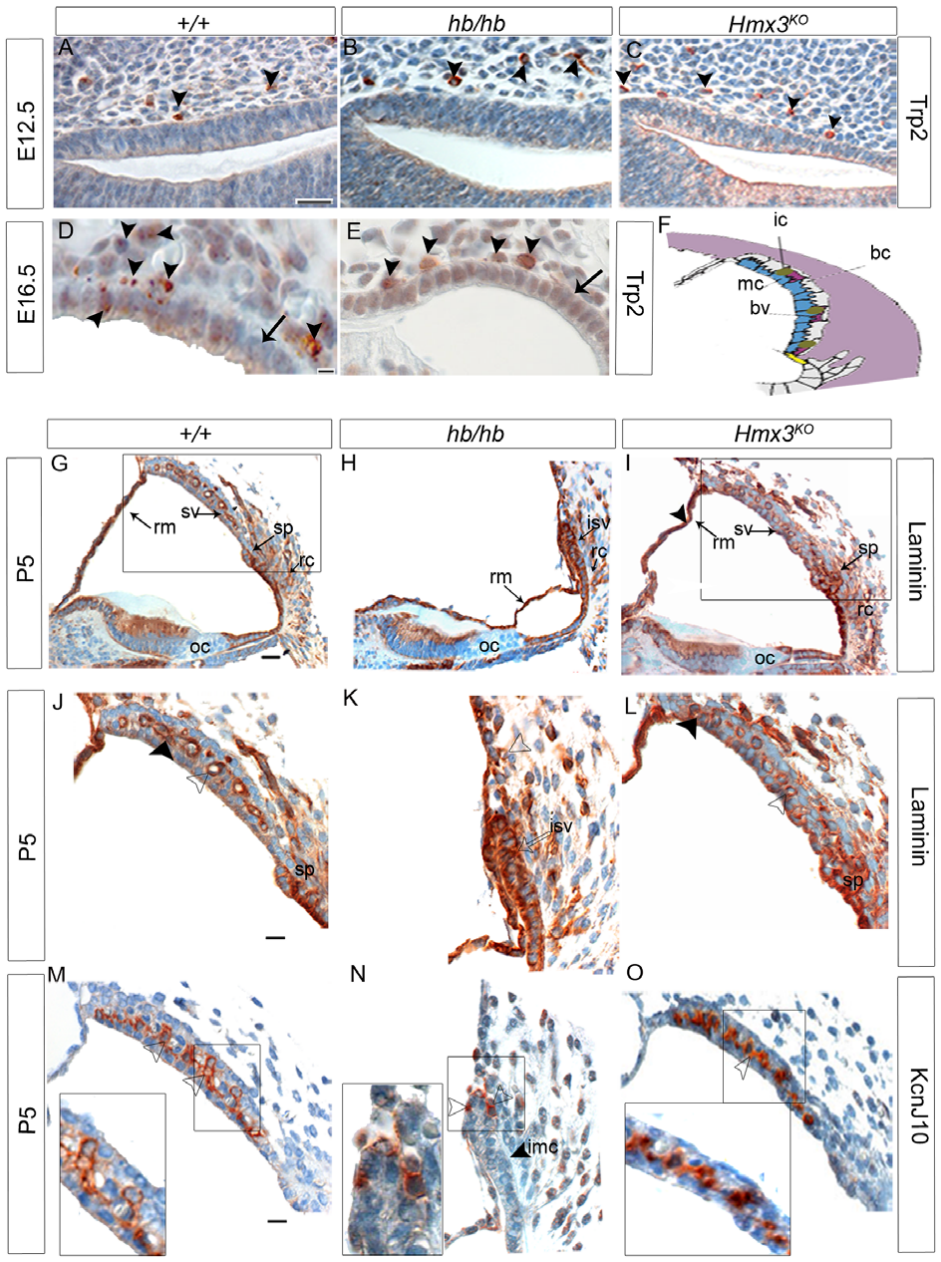
**A–E:** Trp2 expression performed on inner ear sagittal sections in *hb/hb, Hmx3^KO^/Hmx3^KO^* and control littermates at E12.5 and E16.5. **A–C**: Trp2 marks neural crest-derived melanoblasts at E12.5 (arrowheads in A). As shown by the arrowheads in B and C, we detect melanoblasts migrating from the neural crest to developing stria is detected in *hb/hb*, *Hmx3^KO^/Hmx3^KO^*, and littermate controls. D–E: While in control mice at E16.5 some intermediate cells precursors are already in the process of interdigitation in the developing stria vascularis (arrowheads in D), none are in the same process in the *hb/hb* mutants with only a couple of them lying outside the epithelium (arrowheads E). Moreover, Hematoxylin and Eosin counterstaining shows that the epithelium of developing stria looks immature in *hb/hb* compared to littermate controls (arrows in D,E). Scale bars: 10 µm. **F**: Cartoon of the cellular structure of stria vascularis. Adapted from [77]. **G–I**: Immunohistochemistry showing Laminin expression in cochlea and general cochlear morphology of *hb/hb, Hmx3^KO^/Hmx3^KO^* and control littermates at P5. Laminin is expressed in all cochlear basal lamina [63], including stria vascularis, Reissner's membrane, root cell processes and spiral prominence (arrows in G). **J–L**: Laminin expression in stria vascularis of *hb/hb, Hmx3^KO^/Hmx3^KO^* and control littermates at P5. At this stage, we detect basal lamina in blood vessel endothelia (arrowhead in J) and in very small pockets below marginal cells (black arrowhead in J) in control mice. In *hb/hb* we detect a much stronger laminin expression (denser basal lamina) around the immature stria vascularis (arrow in K). Moreover, we observe fewer and smaller blood vessels in *hb/hb* compared to littermate controls (examples of blood vessels are labeled with transparent arrowheads in J,K,L). We did not detect any difference in laminin expression in *Hmx3^KO^/Hmx3^KO^* compared to littermate controls (arrowhead in L). **M–O**: Kcnj10 expression in the stria vascularis of *hb/hb, Hmx3^KO^* mutants and control littermates at P5. Kcnj10 is an inward potassium channel of intermediate cells. We detect only some intermediate cells in *hb/hb* mutants (arrowheads in N) compared to their littermate controls at this stage (M). These intermediate cells are just outside the undifferentiated strial epithelium (the black arrowhead in N points to the immature marginal cell layer in *hb/hb*, see also Figure S1). No difference in Kcnj10 expression is detected in *Hmx3^KO^/Hmx3^KO^* at this stage compared to control littermates (arrowhead in O), consistent with their EP values being close to normal. Boxes delimit regions in higher magnification. Scale bars: A–E: 20 µm; G,I: 10 µm; J–O; 20 µm. bc: basal cells, bv: blood vessels, ic: intermediate cells, imc: immature marginal cells, isv: immature stria vascularis, oc: organ of Corti, rc: root cell processes, Rm: Reissner's membrane, sp: spiral prominence, sv: stria vascularis.

In order to understand whether the lack of melanoblasts in the *hb/hb* stria is due to a problem in their migration from the neural crest to the inner ear or to a possible problem in their survival or differentiation, we used Trp2 as a marker for migratory melanoblasts from neural crest [6], [8]. As shown in Fig. 7A–C, Trp2 staining does not show an obvious difference in the number of melanoblasts migrating to the developing stria in *hb/hb* compared with littermate controls. Moreover, we still detect melanoblasts migrating to the developing stria in *Hmx3^KO^*, suggesting that *Hmx3* is not required for melanoblast migration from the neural crest (Fig. 7C). This observation is consistent with the normal coat color displayed by *hb/hb*. In contrast with normal early migration, we show that some melanoblasts have differentiated at E16.5 in *hb/hb*, but these remain outside the stria vascularis, while we can already observe a group of melanoblasts starting to interdigitate into the developing strial epithelium in control mice (Fig. 7D–E), supporting our observations with Myo7A staining at the same age (Fig. 1L,M). Moreover, hematoxylin-eosin counterstaining of the Trp2 stained sections highlights that the epithelial cells look under-differentiated in *hb/hb*, retaining their cuboidal shape with rounded nuclei, while in control mice cell nuclei are flattening and intermediate cell precursors are entering the developing stria (Arrows in Fig. 7 D,E). We obtained the same results using c-kit as a marker of melanoblast survival at E16.5. [6] (data not shown).

Controlled basal lamina degradation is a key mechanism in morphogenesis of complex organs and is the main molecular event in morphogenesis of both stria vascularis, where marginal cells are derived from the epithelium and sit on a basal lamina [21], and the vestibular system [62]. In this work, we demonstrate that both tissues fail to develop properly in *hb/hb* compared to littermate controls, leading to loss of melanoblast interdigitation into the developing stria vascularis.

We therefore analysed laminin expression as a marker of basal lamina [63] in *hb/hb, Hmx3^KO^* and littermate controls at postnatal day 5. At this stage in control mice, basal lamina underlying the marginal cell layer is almost completely disintegrated to allow melanoblasts to interdigitate into the strial epithelium as well as the extensive formation of marginal cell processes [64]. In fact, we detect basal lamina only in blood vessel endothelia and in very small pockets between marginal and intermediate cells in control mice (Fig. 7 G,J), whereas in *hb/hb*, supporting what we have already seen as early as E16.5, the strial epithelium looks under differentiated in the abnormal mutant stria, with a much stronger laminin expression throughout the lateral wall (Fig. 7K). We also confirmed the abnormal persistence of basal lamina below the epithelial marginal cells in *hb/hb* by transmission electron microscopy of the stria vascularis (Figure S3C). These data suggest a delayed or abnormal differentiation of strial epithelium in *hb/hb* mutants compared to littermate controls. No major differences have been detected with laminin expression in *Hmx3^KO^* compared to littermate controls (Fig. 7I,L), excluding a unique role for *Hmx3* in controlling basal lamina degradation, supporting what has already been reported by [44]. We used the Kcnj10 potassium channel as a marker of intermediate cells [65] at P5, and confirmed that the melanoblasts that survive and differentiate to express Kcnj10 in the *hb/hb* inner ear did not interdigitate into the developing epithelium, since the basal lamina is not broken to allow normal intermediate cell interdigitation. These intermediate cells lie just outside the immature marginal cell layer in *hb/hb*, as shown in Fig. 7N, while a healthy intermediate cell layer can be observed in control mice and *Hmx3^KO^* stria vascularis (Fig. 7O).

**Figure 8:**
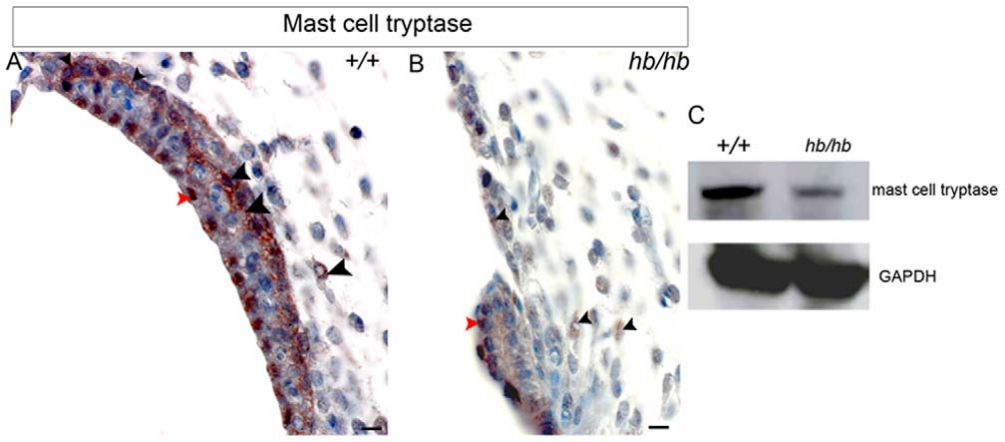
Expression of mast cell tryptase in *hb/hb* and control littermates stria vascularis at postnatal day five. **A,B:** At this stage, our immunohistochemistry detects serine protease expression in proximity of blood vessels and intermediate cells (black arrowheads in A) and nuclei of marginal cells (red arrowhead in A), in line with the mast cell tryptase being involved in DNA synthesis stimulation in some cell types [66]. In *hb/hb* stria we detect reduced protein levels of mast cell tryptase compared to littermate controls, mostly close to its small capillaries (black arrowheads) and marginal cells (red arrowhead). Scale bar: 10 µm. **C:** Extracts from *hb/hb* and control littermate inner ears at P5 were subjected to 10% SDS-PAGE. Western blots were probed with anti-mast cell tryptase antibody. GAPDH is used as loading control.

Finally, we analysed the expression levels of mast cell tryptase, a trypsin-like serine protease that is released by activated mast cells and has been shown to trigger the degradation of extracellular matrix [66], with the aim of checking whether mast cell release of protease could also play a key role in basal lamina digestion in stria vascularis development. We detect decreased levels of mast cell tryptase in *hb/hb* stria vascularis compared to littermate controls (Fig. 8 A,B,C), in line with what was observed in *Chst15* deficient mice in bone marrow-derived mast cells [43].

### Basal lamina degradation is also impaired in *hb/hb* vestibular system

**Figure 9:**
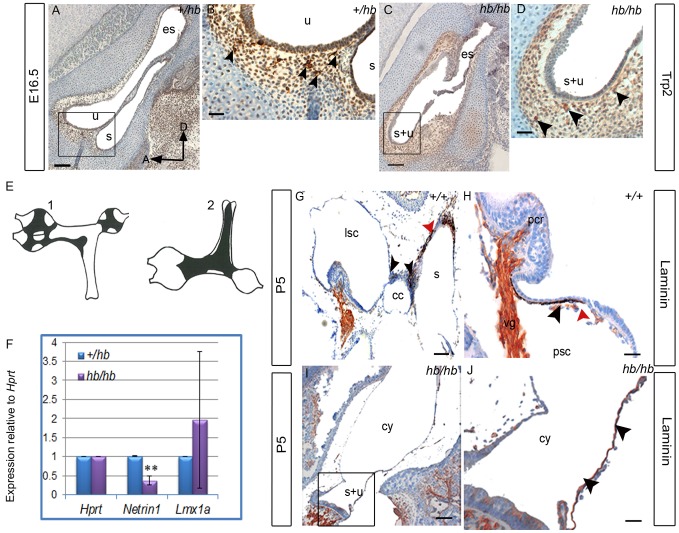
The headbobber stria vascularis remains immature with lack of intermediate cells and impaired basal lamina degradation. **A–D:** Trp2 expression in the vestibular system at E16.5 is analysed by immunohistochemistry in the *hb/hb* and control littermates on sagittal sections. At this stage, melanoblasts have already reached all the different vestibular structures (arrowheads in B, see E). We can still detect amelanoblasts in *hb/hb* vestibular system (arrowheads in D). Scale bars: A,C: 100 µm; B,D: 20 µm. Boxes delimit regions in higher magnification. **E:** Schematic drawings of the flattened vestibular membranes of normally pigmented mice showing distribution of melanocytes in the vestibule viewed on medial (E1) and lateral (E2) position, adapted from [7]. **F:** Quantitative real-time PCR on cDNA generated from RNA from E12.5 littermate embryo half heads. *Netrin-1* mRNA levels are significantly lower in *hb/hb* than in littermate controls. Error bars, s.d. Quantity normalised to *Hprt1* levels. N = 3. **: p<0.01. **G–J:** Laminin staining in the vestibular system of *hb/hb* and littermate controls at P5. In controls, we find melanocytes between the common crus and the other vestibular structures (black arrowheads in G) as well as on the saccular wall (red arrowhead in G) and adjacently to cristae (black arrowhead in H). We show that basal lamina is breaking close to melanocytes (red arrowhead in H). We cannot detect any distinct structure nor melanocytes in the vestibular system of *hb/hb* mutants at this stage (I,J), but we detect the presence of an immature and tight basal lamina all around the vestibular cyst wall, where melanocytes are supposed to be positioned. (black arrowheads in J compared to H). Scale bars: 10 µm. A: anterior, cc: common crus, cy: vestibular cyst, D: dorsal, es: endolymphatic sac, lsc: lateral semicircular canal, pcr: posterior cristae, psc: posterior semicircular canal,,s: saccule, s+u: utriculosaccular space, u: utricle.

We also used Trp2 as a marker of melanoblasts migrating from the neural crest to the vestibular system at E16.5, as well as performed laminin staining at P5 on vestibular system serial sections of *hb/hb* and control littermates. Consistent with what observed in stria vascularis, melanoblast migration from neural crest to the vestibular system appears normal in *hb/hb* compared to littermate controls (Fig. 9A–D). As expected (Fig. 9E), in controls we find melanocytes on boundaries between vestibular structures as well as on the saccular wall and adjacent to cristae (Fig. 9G–H). Moreover, we observed that basal lamina is degraded close to melanocytes suggesting a possible signaling interaction between them. We cannot detect melanocytes in the vestibular system of *hb/hb* mutants at this stage but instead we detect laminin forming an immature dense basal lamina surrounding the fused utriculosaccular space in the *hb/hb* vestibular cyst, where melanocytes would normally be positioned at this stage (Fig. 9I,J). An intermediate scenario is observed in *Hmx3^KO^* vestibular system (data not shown).

Our data suggest a possible role of melanocytes in regulating basal lamina degradation, permitting the normal morphogenesis of stria vascularis as well as vestibular structures.

*Netrin-1*, a member of the laminin-related secreted proteins, is required for the local disruption of basal lamina. It is expressed at high levels in the nonsensory otic epithelium, in cells that will come together to form a fusion plate, a prerequisite for the formation of semicircular canals [62]. Remarkably, the *Netrin1*-positive domains in the wild-type otic vesicle were lost in the double *Hmx2^KO^;Hmx3^KO^* inner ears [45]. We detect a significant reduction of *Netrin-1* mRNA levels in *hb/hb* at E12.5 compared to littermate controls (Fig. 9F). Since no effect on *Netrin-1* expression has been observed in either *Hmx2* or *Hmx3* single mutants, reduced expression of this gene in both the double *Hmx2* and *Hmx3* mutants and headbobber indicates that *Hmx2* and *Hmx3* may function in a mutually redundant way in regulating *Netrin-1* during development, and may act through *Netrin-1* in controlling basal lamina degradation (thus morphogenesis) of the vestibular system.

Finally, one of the *Lmx1a* functional null mutants, dreher (*dr^J^/dr^J^*), shows a similar vestibular phenotype to headbobber [67], so we have investigated whether *Lmx1a* mRNA levels are affected in *hb/hb*. Quantitative RTPCR at E12.5 show extremely variable levels of *Lmx1a* mRNA in *hb/hb* compared to littermate controls (Fig. 9F), and this might be the sign of a mutual transcriptional regulation between *Hmx3*, *Hmx2* and *Lmx1a*, confirming our results from the TRANSFAC analysis.

## Discussion

We found the headbobber combined morphogenetic and cochleoaccular phenotype is due to the insertion of 4 copies in tandem of a transgenic construct on the distal region of mouse Chr7 between *D7Mit105* and *D7Mit12* associated with a 648 kb deletion on Chr7 F3, and this is in line with what reported by Somma et al in [4]. The mutation results in the deletion of *Gpr26*, *Cpxm2* and *Chst15*, as well as disruption of the normal expression of the *Hmx3, Hmx2* and *Nkx1.2* homeobox transcription factors in the inner ear presumably as a consequence of a long-range effect of the mutation on the transcription of neighboring genes. Homozygotes show a severe equilibrium problem starting from two to three weeks of age, with head bobbing and circling and they are completely deaf. The characterisation of the headbobber inner ear phenotype has highlighted a very abnormal structure of the vestibule in *hb/hb* which totally explains the abnormal behaviour, with the absence of all the semicircular canals and the three cristae. The vestibule looks like a simple cyst with the saccule and the utricle fused together in a single utriculosaccular sac and the formation of a single macula. Moreover, despite normal cochlear gross morphology, headbobber homozygotes show a primary cochleo-saccular defect at early postnatal stages, characterised by under-differentiation of stria vascularis and collapse of Reissner's membrane, consistent with what was reported previously [4].

In addition to the data previously published, we show by SEM that in the organ of Corti both inner and outer hair bundles look severely disorganized as early as P5 in *hb/hb*, compared to littermate controls. We have been able to characterise the expression of the three protein coding genes mapping in the headbobber deletion (*Gpr26*, *Cpxm2* and *Chst15*), and show that they all have an interesting expression in the inner ear, which completely fit with the headbobber phenotype. We also use headbobber to gain more insights on the *Hmx2*, *Hmx3* and *Nkx1.2* genetic interactions and transcriptional regulation, and report for the first time a putative role of *Nkx1.2* in inner ear development. Moreover, we show the failure of intermediate cells to integrate into the strial epithelium, associated with complete absence of endocochlear potential. We also analysed the molecular mechanisms behind each aspect of the headbobber phenotype, and find that failure of the basal lamina to regress can explain several key aspects of the pathophysiology and that melanoblasts and mast cells may have a role in this process. Finally, we found a human equivalent of the headbobber mutation, and in this work we review the relevant clinical cases.

### The headbobber deletion

Genetic tools (aCGH and deep sequencing) allowed us to narrow down the headbobber region and discover a big deletion on Chr7 cytoband F3 (Chr7: 139061190–139708657), strongly suggesting this might be the main effect of the transgenic insertional mutation in the headbobber allele, leading to the headbobber inner ear phenotype. These results are in line with our genetic mapping of the phenotype and the transgene, and with the results reported previously [4]. The deleted region contains three protein coding genes: 1) *Gpr26*, an orphan G-coupled receptor; 2) *Cpxm2*, a carboxypeptidase predicted to act as a binding protein; 3) *Chst15*, a carbohydrate sulfotransferase. For the first time, we report their expression in the murine inner ear and we link them to both mouse and human deafness. In detail, *Gpr26* is expressed in the spiral ligament after birth, and recycling potassium though the fibrocyte network is one of several processes that provide potassium to intermediate cells of the stria vascularis, an essential process for normal cochlear function [68], [69]. *Cpxm2* shows specific expression in the intermediate cells of stria vascularis at P5 which fits with the intermediate cell phenotype observed in *hb/hb* as early as E16.5 and subsequent absence of endocochlear potential. Finally, we detect *Chst15* expression in the basal region of stereocilia and cell body of both inner and outer hair cells as well as in marginal cells and their interdigitations in stria vascularis. This is consistent with both the stereocilia disorganisation- showing fusion at their base - and the strial developmental failure observed in the homozygote mutants. Taken together, our results show that in this particular region of Chr7 there is an interesting group of genes playing an important role in stria vascularis development and auditory function. Their absence, together with the reduced expression of *Hmx3, Hmx2* and *Nkx1.2*, which are located at the same chromosomal region, causes deafness and vestibular problems both in mice and humans.

### Headbobber is an hypomorphic mutation of *Hmx3* and *Hmx2*

*Hmx3* lies about 8 kb away from *Hmx2* on Chr7, and the striking similarity in their expression patterns and in the sequence of their homeodomains, as well as their close proximity on the chromosome, raises the possibility that *Hmx2* and *Hmx3* may share common regulatory elements and have overlapping developmental functions, as previously discussed [46]. Our hypothesis is that the transgene has disrupted their regulatory elements and that is consistent with many observations: 1) The insertion of the *hb* transgene causes a deletion of about 648 kb in the distal part of chromosome 7F3, approximately 375 kb telomeric to the *Hmx3* and *Hmx2* locus; 2) We confirmed by Southern blot and DNA sequencing that the coding sequences of *Hmx3* and *Hmx2* are physically present on Chr7 in *hb/hb* mice; 3) Low *Hmx3* and *Hmx2* mRNA levels have been detected in *hb/hb* when compared to levels observed in the littermate controls at E12.5 (in line with what reported in [4]); and 4) A complementation test was carried out between headbobber homozygotes and the *+/Hmx3^KO^* mice, with the compound heterozygotes showing circling behaviour and intermediate vestibular abnormalities as well as endocochlear potential values, which suggests that downregulation of *Hmx3* may contribute to the headbobber phenotype.

The headbobber vestibular phenotype is more severe than the one displayed by either the *Hmx2* or *Hmx3* single knockouts and these observations are in complete accordance with the suggestion of their redundant function in the inner ear [46]. On the other hand, the headbobber vestibular phenotype is less severe than the one displayed by the *Hmx3/Hmx2* double knockout, which does not develop any vestibular system [45], which is consistent with the headbobber mutation being hypomorphic for *Hmx2* and *Hmx3*.

### A new putative role for *Nkx1.2* in inner ear development

Our results show the downregulation and, for the first time, the potential involvement of another member of the *Hmx* family of transcription factors in the headbobber phenotype: *Nkx1.2*, which might share regulatory elements with *Hmx3* and *Hmx2* on Chr7. This gene has already been reported to be transiently expressed in the developing central nervous system [48] and in this work we describe its expression in the developing as well as in the postnatal stria vascularis; in fact, its expression shifts from prospective intermediate cells during development to marginal cells and their projections at postnatal stages. At P5, *Nkx1.2* is also expressed in organ of Corti (inner and outer hair cells) and vestibular maculae. Therefore, we can hypothesise for the first time a role for *Nkx1.2* in inner ear development and of particular interest is its potential role in controlling melanoblast survival (see further discussion).

### Potential interactions suggested by the TRANSFAC analysis

We found that *Hmx2, Hmx3* and *Nkx1.2* themselves, together with many other homeobox transcription factors with known function in inner ear development such as *Emx2*, *Hoxa2*, *Otx1*, *Otx2*, *Lmx1a*, *Lmx1b*, *Pax2*, *Pax6*, *Six1* and *Six2*, are predicted to recognise high affinity binding sites in the deleted sequence. These analyses suggest that they might play a role in the regulation of *Hmx3*, *Hmx2* and *Nkx1.2* transcriptional levels. *Six1* has already been shown to repress *Hmx3* and *Hmx2* expression in the ventral part of the otocyst [70], although *Pax2* deficient mice do not show any change in either *Six1* or *Hmx3* expression levels [71]. This observation suggests that *Six1* and *Hmx3* (but not *Pax2*) may act in the same transcriptional pathway, with *Six1* acting upstream of *Hmx3* and *Hmx2*. Moreover, both the *Otx1* and the *Otx2* deficient mice show an incomplete separation of the utricle and saccule [72] similar to the *Hmx3* and *Hmx2* knockouts, suggesting that they might also act in the same pathway. Finally, our quantitative RT-PCR shows a possible mutual transcriptional regulation between *Hmx3*, *Hmx2* and *Lmx1a*.

### The headbobber mutation affects early crista specification but does not affect early hair cell differentiation

While the function of the *Hmx* genes in the inner ear has been extensively studied, very little is known about their genetic interactions. Headbobber might be a good model to study the role of *Hmx3*, *Hmx2* and *Nkx1.2* in inner ear development and place them in a cascade of events. To this purpose, the expression of genes with a demonstrated role in inner ear development has been examined on wild-type and *hb/hb* inner ear sections at different embryonic stages.

Analysis of hair cell differentiation using Sox2 (marker for the prosensory regions), Calretinin (marker for the developing hair cells), p27^Kip1^ (sensory regions), and Jag1 (mature supporting cells, data not shown) showed no overt alteration in their expression in the remaining sensory patches of the *hb/hb* inner ear. This suggests a normal spatio-temporal development of the only two prosensory and sensory regions that form in the *hb/hb* mice, which are the fused maculae and the organ of Corti. So we can conclude that when a patch forms, it can fully differentiate. Either the lack of a gene regulatory relationship between the three homeobox genes and the genes involved in the development of sensory patches or the inability of these three genes alone to alter the regulatory cascade may account for these findings.

We reveal a possible interaction between *Hmx3, Hmx2*, *Nkx1.2* and the bone morphogenetic protein 4 (*Bmp4*) by detecting the *Bmp4* RNA in *hb/hb* mice. At E12.5, during fusion plate formation, *Bmp4* transcripts are detected in the developing cristae of wild type mice and are absent in the fusion plate. However, despite the absence of recognizable cristae in *hb/hb Bmp4* transcripts were found in two regions of the vestibular system in *hb/hb* embryos, at this stage. Further expression analysis of *Bmp4* expression in *hb/hb* at E16.5 failed to detect *Bmp4* transcripts in the vestibular system at this later stage (data not shown). These observations suggest that in *hb/hb* the cristae might be specified initially but fail to develop properly in the absence of *Hmx* genes, perhaps meaning that *Bmp4* alone is not able to specify a prosensory region (crista). *Hmx3* and *Hmx2* might regulate the spatio-temporal expression of *Bmp4*, possibly acting on its promoter regions [36]. As we detected *Hmx2*, *Hmx3* and *Bmp4* transcripts in the non-sensory epithelial cells surrounding cristae in control mice, one might suppose that their interaction is crucial to control sensory cell fate determination. As we have detected a downregulation of *Netrin-1* expression in *hb/hb* compared to littermate controls, we can conclude that *Hmx3, Hmx2* and *Nkx1.2* might play a role in controlling *Netrin-1* transcription in the inner ear and therefore we suggest that *Netrin-1* might be a downstream target of *Hmx* genes in the vestibular system.

### A proposed mechanism to explain both the hearing and vestibular phenotypes shown by the headbobber mutant

Controlled basal lamina degradation is a key mechanism in morphogenesis of complex organs such as the vestibular system and stria vascularis in the inner ear. Although still little is known about its molecular mechanisms, mouse models have given important insights, and we now know for example that *Netrin-1* and *Hmx2* are required for the local disruption of the basal lamina by acting in epithelial-mesenchymal signalling during semicircular canal formation [44], [62]. We propose the impairment of the normal process of degradation of the basal lamina as an explanation for both deafness and the vestibular phenotype displayed by headbobber homozygous mutants. Controlled basal lamina degradation occurs starting from E12.5 in vestibular system development (at the canal pouch stage) [73], and this is exactly when we can start detecting the *hb/hb* vestibular abnormality. On the other hand, controlled basal lamina degradation to allow cytodifferentiation in strial epithelium with the initial interdigitation of intermediate cells, followed by formation and extensive elongation of marginal cell processes, happens later in development. By postnatal day five the basal lamina underlying the marginal cell layer is almost completely disintegrated [25].

In fact, we detect abnormalities in strial epithelium differentiation starting as early as E16.5 in *hb/hb* mutants, which is the stage at which we observe the lack of prospective intermediate cells entering and interdigitating into the strial epithelium. Although specific molecular targets for the *Hmx* genes in the stria vascularis are unknown, they could affect intermediate cells (melanocytes, where *Hmx2* and *Hmx3* are expressed at postnatal stages). We report that *Nkx1.2* (downregulated in *hb/hb*) is expressed in prospective intermediate cells at E16.5 and in marginal cells after birth, thus it might also have a role in controlling basal lamina degradation in the stria vascularis. In addition to this, *Cpxm2*, deleted in *hb/hb*, shows expression in the intermediate cells at postnatal stages. We observe lack of melanocytes in both vestibule and cochlea in *hb/hb* but we have demonstrated that this phenomenon is not due to an impaired migration of melanoblasts from the neural crest: in *hb/hb* mutants, melanoblasts migrate normally to the vestibular system and the stria vascularis but then they are not able to interdigitate into the developing epithelia. In stria vascularis the epithelial marginal cells remain in the immature cuboidal state when they should start interdigitating with intermediate cells. We hypothesize that, as a direct consequence of the failure of their tissue interdigitation, the *hb/hb* melanoblasts might die or de-differentiate into a non-recognizable form and this would explain lack of melanocytes in the inner ear several days after neural crest migration.

Our data suggest a possible role of melanocytes in regulating basal lamina degradation and therefore of normal morphogenesis of stria vascularis as well as vestibular structures. For example, it is evident that in a mature vestibular system melanocytes are present between different structures (see cartoon in Fig. 9), and this might be an indication of their active role in vestibular system morphogenesis and explain why the *hb/hb* vestibular system, which completely lacks recognizable mature melanocytes, is lacking of any evident vestibular structure and shows a tight basal lamina surrounding a vestibular cyst instead.

Of course, our data might suggest either a key role for melanocytes in basal lamina breaking or, vice versa, a key role for basal lamina in driving melanoblast integration in the developing epithelium. The answer to this question can be found in the literature: it has been previously demonstrated that in viable dominant spotting, a deaf mouse mutant with impaired survival of melanoblasts when they reach the inner ear, basal lamina degradation in stria vascularis is impaired [7], [8], [25], [64], [74]. However, *viable dominant spotting* mutant mice also lack mast cells [75] and we also report that mast cell tryptase protein levels are reduced in *hb/hb* stria vascularis compared to littermate controls, in line with what has previously been observed in the hematopoietic cell lines in *Chst15^KO^* [43]. Since mast cell tryptase is already reported to be involved in extracellular matrix degradation [66], we conclude that: 1) Mast cells tryptase might be involved in the digestion of basal lamina in stria vascularis, 2) Chst15 might contribute to mast cell protease levels 3) Melanoblasts (perhaps mechanically) and mast cells (enzymatically through release of protease) may act in parallel in controlling stria vascularis development and basal lamina degradation. Gaining more insight into this mechanism could have important implications in future treatments for human prenatal deafness, as attempts to reconstitute the mast cell population in the *viable dominant spotting* mutants have already been made [76].

### Headbobber and human deafness

Different-sized deletions of the chromosomal region in humans which is orthologous to the headbobber region (10qter) have been reported in fifty-nine patients with common features including behavioral problems, prominent nose, facial asymmetry, growth retardation, severe mental retardation, cardiac abnormalies, digital malformation, malformed ears, and, in the case of nineteen of them sharing a common deletion, sensorineural hearing loss and vestibular problems. The first patient with partial deletion of the long arm of chromosome 10 was described in 1978 [14] and then the number of reported clinical cases has been exponentially increasing, so that ten years later Wulfsberg et al proposed that a 10q-syndrome existed [15]. The deletions' breakpoints range from 10q23.3 to q26.3 and the reported cases include de novo and familial, interstitial deletions and translocations involving only the terminal band of chromosome 10 [16], [17]. Wulfsberg et al also proposed that there was a positive correlation in the severity of the malformation with increasing size of the chromosome deletion; however, only the improvement of the banding techniques has allowed better localising of the exact breakpoints and identifying of candidate genes for the different phenotypes showed by the patients. For example, Irving et al observed that FGFR2 was deleted in eight of their 10q deletion cases, all showing facial asymmetry, suggesting for the involvement of this gene in the specific phenotype. Moreover, three of their cases with terminal deletions show behavioral problems and in all of them the C-terminal binding protein 2 (CTBP2) and Calcycon (D1 dopamine receptor interacting protein) are deleted. One of them, showing a bigger deletion covering part of the telomeric region and involving the headbobber genes (10q25.2–26.1), was also diagnosed with high frequency sensorineural hearing loss [17].

**Figure 10:**
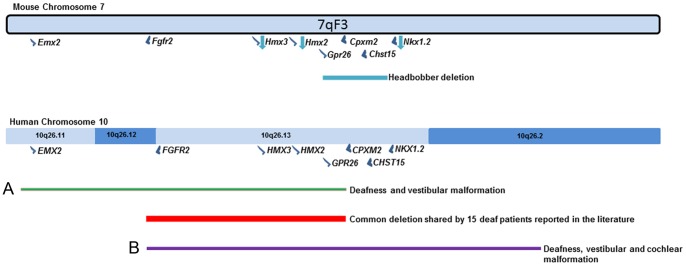
Overview of the headbobber deletion and 10q deletion patients. Blue arrows pointing down symbolize downregulated genes. Adapted from [18].

Of particular relevance for our study, in 2009 Miller et al reviewed all the clinical cases of 10qter syndrome with sensorineural hearing loss, and reported four unrelated patients with de novo, overlapping deletions of the long arm of chromosome 10 [18]. Two of them showed profound, sensorineural deafness as well as an abnormally enlarged cystic vestibule detected by CT imaging in both patients (Fig. 10A,B), leading to delayed walking. These two patients share a common deletion (2.5 Mb, q26.12–q26.13) with fifteen deaf patients previously reported in the literature [16], [17], although the size of the deletions detected in each individual patient varies, spanning from 10q25.3 to 10q26.3 (Chr10: 115,000,000–135,000,000), and this is consistent with the variety of different morphological and behavioural phenotypes they show beside the hearing loss. Interestingly, genes like *FGFR2*, *HMX3*, *HMX2* and *GPR26* map in the common deleted region. *Fgfr2* expression is not affected in the homozygous headbobber mutant, whereas *Hmx2* and *Hmx3* are downregulated and *Gpr26* is absent. Therefore, our hypothesis is that *Hmx3* and *Hmx2* have a role in causing the vestibular phenotype accounting for the vestibular morphological defects reported both in mice and humans, while *Gpr26* might be required for normal hearing function. One of the two patients, however, uniquely shows cochlear abnormalities (Mondini-type) such as a narrowed internal auditory canal, a shorter, rudimentary shaped cochlea as well as an enlarged cochlear aqueduct, together with vestibular abnormalities and speech delay (Fig. 10B). This patient's 10qter deletion affects also *CPXM2*, *CHST15* and *NKX1.2*, supporting their putative role in controlling inner ear morphogenesis both in human and mouse by acting in parallel through melanoblasts and melanocytes (*Nkx1.2* and *Cpxm2*), marginal cells (*Chst15* and *Nkx1.2*) and mast cells (*Chst15*). Detailed analysis of the hearing phenotypes of the single knockouts for the above genes in the mouse will highlight their single contributions to the complex syndromic manifestations in human 10qter syndrome.

## Supporting Information

Figure S1 **Genetic mapping of the headbobber mutation and transgenic insertion on mouse Chr7, between markers** ***D7Mit105*** **and** ***D7Mit12*** **in a region of 8.01 Mb.** **A:** Haplotype diagram of the headbobber backcross mice. The numbers represent the number of mice that have the indicated haplotype. A black box has been used to represent a headbobber allele at a given locus and a white box to represent a CBA/Ca allele ***B*** **:** Haplotype diagram of the transgene-mapping backcross. The numbers represent the number of mice that have the indicated haplotype. A black box has been used to represent the presence of the transgenic insertion at a given locus and a white box to represent a CBA/Ca allele and a lack of the insert. **C:** The graph shows the average neo count per chromosome of 34 *hb* mice from 8 different mating pairs. The mice fall into 3 distinct groups, mice 1–4 show a neo count of 0, mice 5–22 show an average count of 2.05 and mice 23–34 show an average count of 4.12. Mouse genotype has been confirmed through either short range PCR or phenotype/ear morphology, mice 1–4 are confirmed WT, 5–22 confirmed heterozygotes and 23–34 confirmed homozygotes. This would indicate a neo count of 0 in the WTs, 4 in the heterozygotes and 8 in the homozygotes.
(TIF)

Figure S2 **A–B:** Hmx3 expression in intermediate cells of stria vascularis at P5. In B, Kcnj10 has been used as a marker of intermediate cells on an adjacent section to A (arrowheads). Scale bar: 5 µm; **C–D:** Expression analysis of Kcnq1, marker of marginal cells of stria vascularis, in *hb/hb* and littermate controls at P5 (black arrowheads). Scale bar: 10 µm. **E–F**: Immunohistochemistry for Fgfr2 at P5 in *hb/hb* and littermate controls. At this stage, Fgfr2 is located in hair cells (black arrowheads) and tectorial membrane (red arrowhead). No significant differences in the Fgfr2 protein levels are detected in *hb/hb* mutants compared to littermate controls. Scale bar: 10 µm. **G–I:** 3D reconstruction of the endolymphatic compartments of newborn *hb/hb, hb/Hmx3^KO^* and control littermates, showing the cyst-like vestibular structure observed in *hb/hb* and *hb/Hmx3^KO^*.
(TIF)

Figure S3 **Transmission electron microscopy of stria vascularis of** ***hb/hb*** **(B,C) and control littermates (A) at 14 months, showing the collapse of Reissner's membrane, the loss of the normal three cell layers organisation and of the cell-cell interdigitations in** ***hb/hb*** **.** Moreover, the arrowheads in C point to the abnormal basal lamina below the epithelial marginal cells in *hb/hb*. Scale bar: 5 µm. bc: basal cells, bl: basal lamina, bv: blood vessel, ic: intermediate cells, int: interdigitation, mc: marginal cell, Rm: Reissner's membrane, sv: stria vascularis.
(TIF)
