# Supplementary figures and images for: Headbobber: A Combined Morphogenetic and Cochleosaccular Mouse Model to Study 10qter Deletions in Human Deafness
Source: PLoS One. 2013 Feb 14;8(2):e56274. doi: 10.1371/journal.pone.0056274 (PMC3572983; doi:10.1371/journal.pone.0056274)

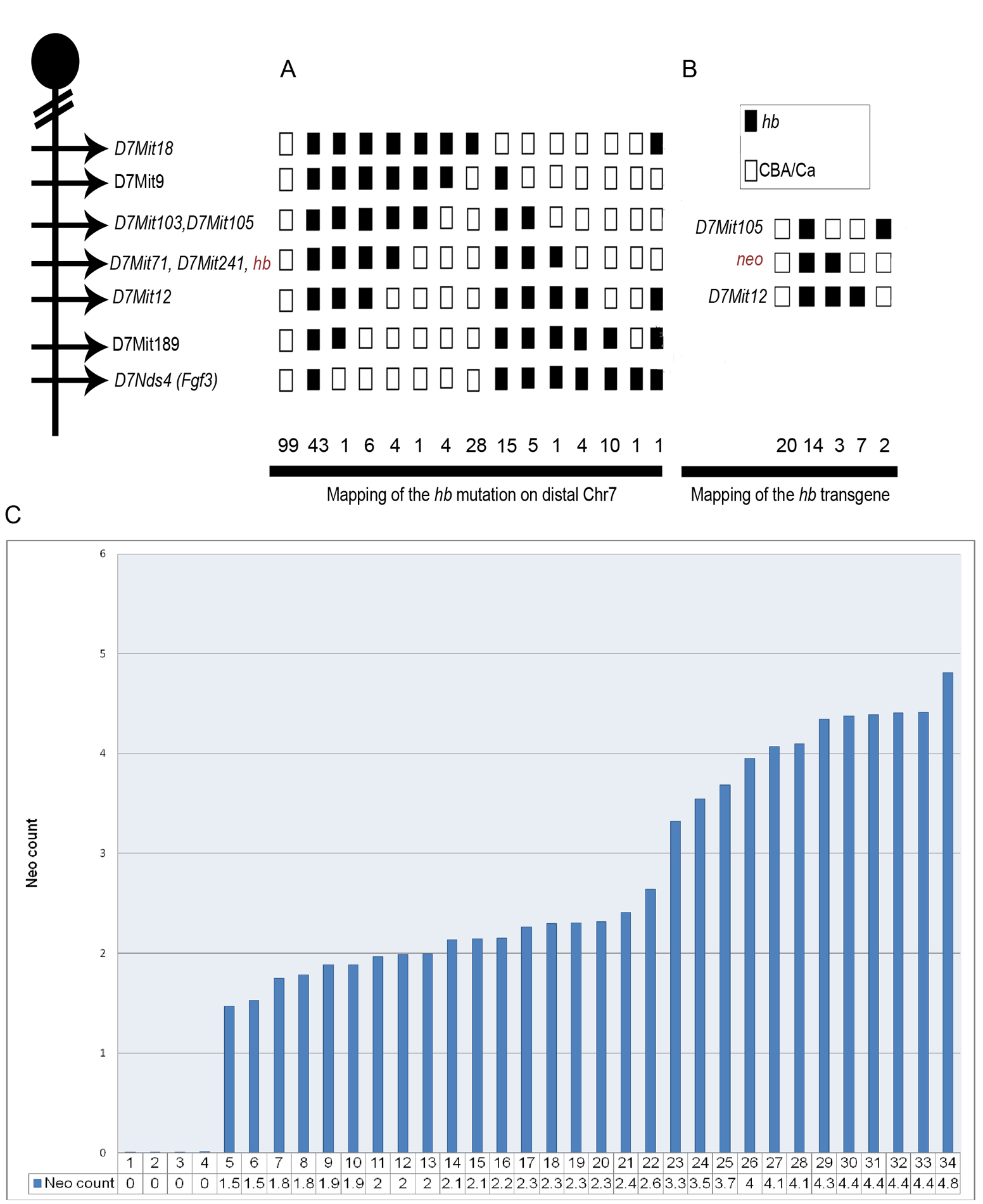

Supplement: Figure S1 — Genetic mapping of the headbobber mutation and transgenic insertion on mouse Chr7, between markers D7Mit105 and D7Mit12 in a region of 8.01 Mb. A: Haplotype diagram of the headbobber backcross mice. The numbers represent the number of mice that have the indicated haplotype. A black box has been used to represent a headbobber allele at a given locus and a white box to represent a CBA/Ca allele B : Haplotype diagram of the transgene-mapping backcross. The numbers represent the number of mice that have the indicated haplotype. A black box has been used to represent the presence of the transgenic insertion at a given locus and a white box to represent a CBA/Ca allele and a lack of the insert. C: The graph shows the average neo count per chromosome of 34 hb mice from 8 different mating pairs. The mice fall into 3 distinct groups, mice 1–4 show a neo count of 0, mice 5–22 show an average count of 2.05 and mice 23–34 show an average count of 4.12. Mouse genotype has been confirmed through either short range PCR or phenotype/ear morphology, mice 1–4 are confirmed WT, 5–22 confirmed heterozygotes and 23–34 confirmed homozygotes. This would indicate a neo count of 0 in the WTs, 4 in the heterozygotes and 8 in the homozygotes. (TIF) [file pone.0056274.s001.tif]

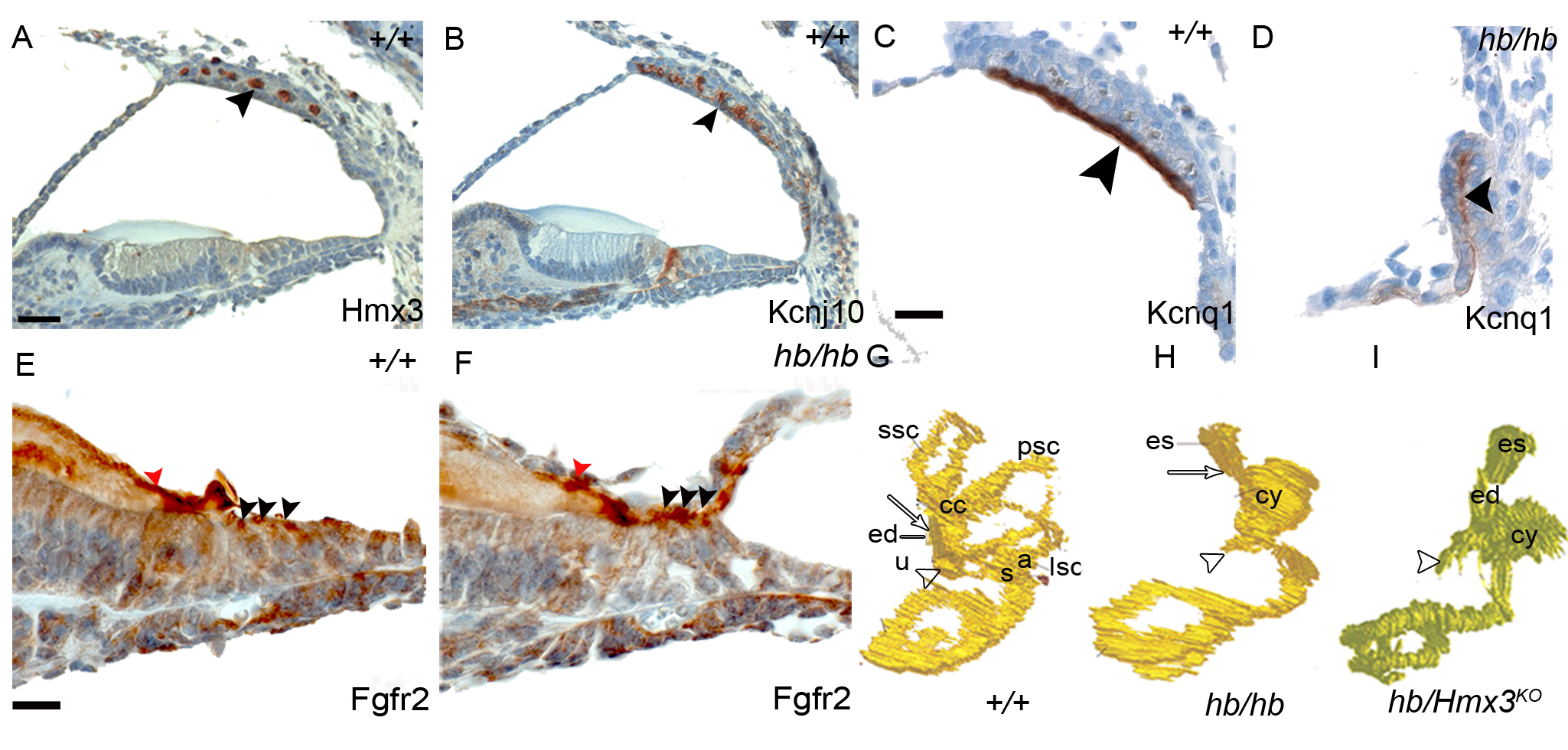

Supplement: Figure S2 — A–B: Hmx3 expression in intermediate cells of stria vascularis at P5. In B, Kcnj10 has been used as a marker of intermediate cells on an adjacent section to A (arrowheads). Scale bar: 5 µm; C–D: Expression analysis of Kcnq1, marker of marginal cells of stria vascularis, in hb/hb and littermate controls at P5 (black arrowheads). Scale bar: 10 µm. E–F: Immunohistochemistry for Fgfr2 at P5 in hb/hb and littermate controls. At this stage, Fgfr2 is located in hair cells (black arrowheads) and tectorial membrane (red arrowhead). No significant differences in the Fgfr2 protein levels are detected in hb/hb mutants compared to littermate controls. Scale bar: 10 µm. G–I: 3D reconstruction of the endolymphatic compartments of newborn hb/hb, hb/Hmx3KO and control littermates, showing the cyst-like vestibular structure observed in hb/hb and hb/Hmx3KO. (TIF) [file pone.0056274.s002.tif]

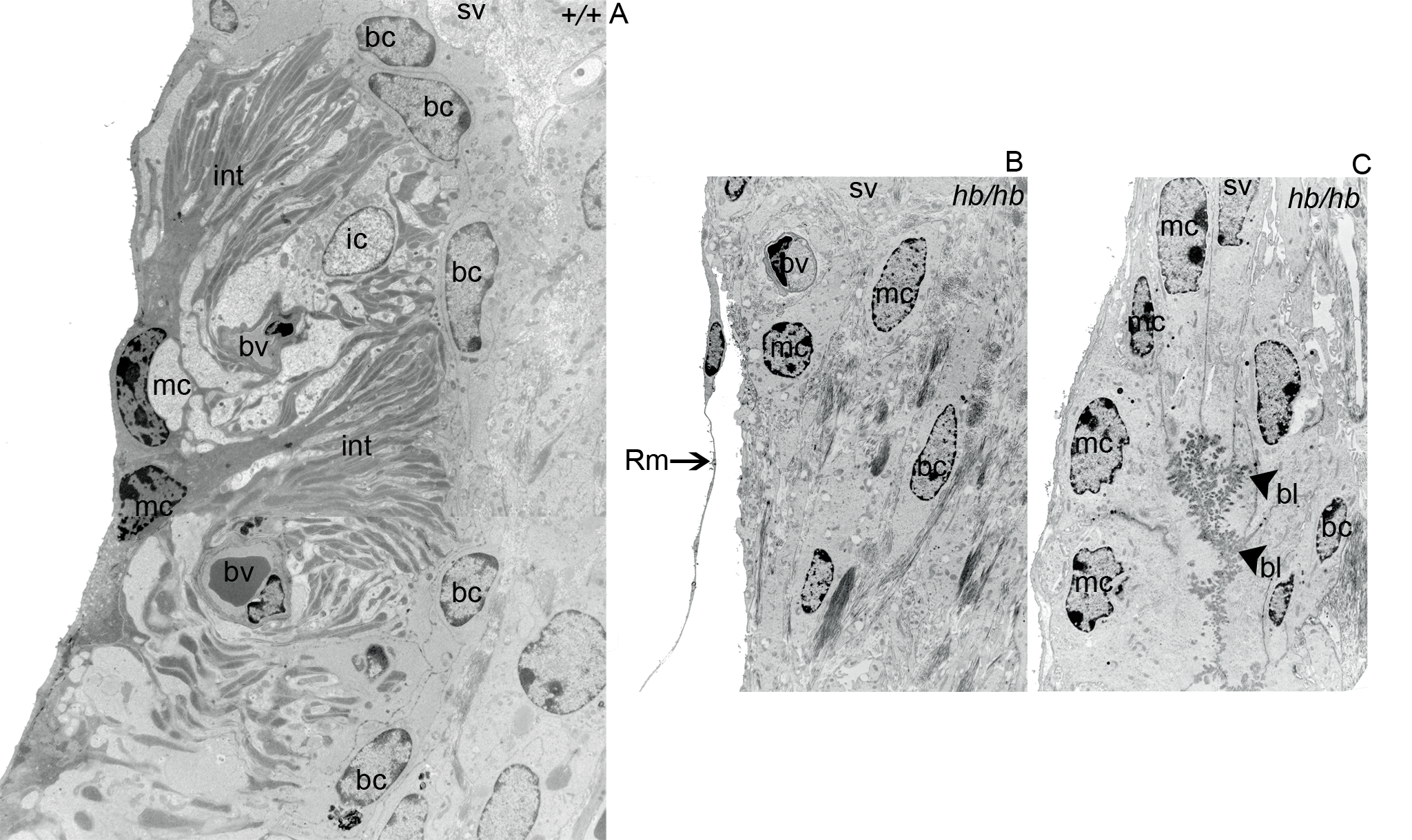

Supplement: Figure S3 — Transmission electron microscopy of stria vascularis of hb/hb (B,C) and control littermates (A) at 14 months, showing the collapse of Reissner's membrane, the loss of the normal three cell layers organisation and of the cell-cell interdigitations in hb/hb . Moreover, the arrowheads in C point to the abnormal basal lamina below the epithelial marginal cells in hb/hb. Scale bar: 5 µm. bc: basal cells, bl: basal lamina, bv: blood vessel, ic: intermediate cells, int: interdigitation, mc: marginal cell, Rm: Reissner's membrane, sv: stria vascularis. (TIF) [file pone.0056274.s003.tif]
